# Modelling Coral Reef Futures to Inform Management: Can Reducing Local-Scale Stressors Conserve Reefs under Climate Change?

**DOI:** 10.1371/journal.pone.0080137

**Published:** 2013-11-18

**Authors:** Georgina G. Gurney, Jessica Melbourne-Thomas, Rollan C. Geronimo, Perry M. Aliño, Craig R. Johnson

**Affiliations:** 1 Australian Research Council Centre of Excellence for Coral Reef Studies, James Cook University, Townsville, Queensland, Australia; 2 School of Zoology, University of Tasmania, Hobart, Tasmania, Australia; 3 Australian Antarctic Division, Department of Sustainability, Environment, Water, Population and Communities, Kingston, Tasmania, Australia; 4 Antarctic Climate & Ecosystems Cooperative Research Centre, University of Tasmania, Hobart, Tasmania, Australia; 5 Conservation International Philippines, Quezon City, Philippines; 6 Marine Science Institute, University of the Philippines, Quezon City, Philippines; 7 Institute for Marine and Antarctic Studies, University of Tasmania, Hobart, Tasmania, Australia; Leibniz Center for Tropical Marine Ecology, Germany

## Abstract

Climate change has emerged as a principal threat to coral reefs, and is expected to exacerbate coral reef degradation caused by more localised stressors. Management of local stressors is widely advocated to bolster coral reef resilience, but the extent to which management of local stressors might affect future trajectories of reef state remains unclear. This is in part because of limited understanding of the cumulative impact of multiple stressors. Models are ideal tools to aid understanding of future reef state under alternative management and climatic scenarios, but to date few have been sufficiently developed to be useful as decision support tools for local management of coral reefs subject to multiple stressors. We used a simulation model of coral reefs to investigate the extent to which the management of local stressors (namely poor water quality and fishing) might influence future reef state under varying climatic scenarios relating to coral bleaching. We parameterised the model for Bolinao, the Philippines, and explored how simulation modelling can be used to provide decision support for local management. We found that management of water quality, and to a lesser extent fishing, can have a significant impact on future reef state, including coral recovery following bleaching-induced mortality. The stressors we examined interacted antagonistically to affect reef state, highlighting the importance of considering the combined impact of multiple stressors rather than considering them individually. Further, by providing explicit guidance for management of Bolinao's reef system, such as which course of management action will most likely to be effective over what time scales and at which sites, we demonstrated the utility of simulation models for supporting management. Aside from providing explicit guidance for management of Bolinao's reef system, our study offers insights which could inform reef management more broadly, as well as general understanding of reef systems.

## Introduction

Coral reefs are of immense natural and anthropocentric value. Aside from boasting the highest diversity of all marine ecosystems [1], coral reefs provide a wealth of ecosystem goods and services to millions of people in more than 100 tropical countries [2], [3]. However, coral reefs are in decline worldwide [4], [5], and given the present momentum of human population growth, resource use and the imminent threat of climate change, this decline is likely to be exacerbated [6]. Coral reef degradation is driven by a myriad of stressors, which are largely a result of marine- and land-based anthropogenic activities such as fishing, coastal development, and aquaculture [7]. More recently, climate change has emerged as a principal threat to coral reefs [8], [9]. There is growing evidence that the vulnerability of coral reefs to the effects of climate change is increased by chronic, local-scale stressors [10], [11] and that the cumulative effect of these stressors may be synergistic [12], [13]. Here, a stressor is defined as any environmental or biotic factor that exceeds natural levels of variation to cause a harmful effect on a system or organism [14]–[16].

### Managing coral reefs under climate change

Given that climate change impacts on coral reefs cannot be mitigated directly, the question arises whether reduction of stressors that originate and can be managed at a local scale (i.e. local-scale stressors) provides a tractable opportunity to increase the potential of coral reefs to cope with inevitable changes in the climate [2], [11]. Critical to undertaking such management actions is an understanding of how reef systems will respond to alternative actions under different climatic scenarios. Managers need to be able to identify appropriate management actions from a suite of options, and have an understanding of where they will most likely be effective and over what time scales. Although the management of local-scale stressors, typically fisheries and poor water quality (i.e. nutrification and sedimentation), is widely advocated to conserve coral reefs in the context of climate change [2], [7], the extent to which reduction of local-scale stressors confers resilience to climate change and how to identify the particular stressors that should be targeted by management to most cost-effectively optimise resilience, is uncertain [17], [18]. These issues have been flagged as key research questions for reef scientists [13].

Inherent to assessing the importance of local management action is knowledge of the combined impact of multiple stressors [19], [20], including the nature of their interactions; i.e. whether the effect of multiple stressors is simply the sum of the effects of each stressor (additive), or if the cumulative effect is more than (synergism) or less than (antagonism) the additive effect [20], [21]. Although stressors almost always co-occur [6] and the nature of their interaction can have a profound influence on management outcomes [19], research and management has tended to consider stressors in isolation [14], [20]. Thus there is limited understanding of the effect of concurrent stressors on ecological systems in general [20], [22], and marine systems in particular [23], [24], which hampers managers' ability to diagnose drivers of environmental degradation and consequently the appropriate course of management action. The dearth of research on stressor interactions is likely because isolating and quantifying individual effects and interactive effects is a difficult task [6], [15]. This is particularly pertinent in natural systems where most stressors occur simultaneously; thus, the few existing studies of multiple stressors tend to be manipulative experiments at small spatial scales [14], [20].

### Modelling coral reef systems

Models are ideal tools for tackling questions regarding cumulative stressors at appropriate spatial and temporal scales, and for supporting environmental decision-making in general, through elucidating the consequences of alternative management actions in the context of global climate change [25]. Stochastic simulation models are particularly relevant because they can be used to explore the possible outcomes of alternative management scenarios in the face of imperfect knowledge of the future, and with a limited understanding of complex ecological systems [26], [27]. This is valuable given that decisions about when, where and how management actions should be implemented are founded on our predictions of the future [28]. In addition, models provide a useful way in which to distil the intrinsic complexity of coral reef systems, and they allow the ready application of complex experimental designs to address critical management questions at different temporal and spatial scales, including those related to the effects of simultaneous stressors.

Models have been used widely to aid understanding of the impact of stressors on coral reefs [29], [30], including analysis of reef state under alternative climatic [8] and/or management scenarios [31], [32]. However, there are a number of key limitations of existing models with respect to their ability to address questions relevant to management. First, a whole-of-system approach is not always adopted. For example, some studies consider the dynamics of corals [30], [33] or fish [32] only, which does not allow for investigation of whole-of-system processes, such as phase-shifts from coral- to macroalgal-domination. Other studies do not explicitly model consumer dynamics [8], [31], which prevents investigation of feedbacks between the benthos and consumers. A second important limitation is that existing models are often not instantiated using spatially explicit data on reef state for particular locations, instead they tend to be designed to apply to a general system within a broad bio-geographical area, most commonly the Caribbean [29], [31], [34]. Third, modelling studies tend to be restricted to examining the impact of one or two stressors. For example, scenario analyses have focused solely on fisheries [32], [35], the effects of climate change [8], or water quality [36], and only a handful examine the effects of two concurrent stressors [34], [37]. Thus there is a scarcity of spatially explicit models able to provide decision support for management of the multiple simultaneous stressors that many coral reefs are subject to, including how management of local stressors might affect future reef state under the expected effects of climate change.

Recent work by Melbourne-Thomas et al. [38], [39] is an exception; these studies used models instantiated at a regional scale for particular sites to examine the effect of multiple stressors on coral reef systems. While consideration of regional-scale dynamics (e.g. connectivity between reefs through dispersal of fish larvae) is critical for the effective management of coral reefs, equally important is an understanding of reef systems at a local scale. Reef state and the magnitude of stressors will differ spatially even at a local scale, requiring management actions to be tailored to the local context. Further, governance and management of marine resources is undertaken locally in many developing countries [40]. However, studies using simulation models of coral reefs as a decision support tool for local-scale management of multiple stressors are lacking.

### Aims

Here we use a simulation model to investigate the extent to which management of local-scale stressors might influence future trajectories of coral reefs under varying climatic scenarios. We use a model originally developed by Fung et al. [41], which captures the dynamics of key coral reef consumers and benthic organisms. We validate it for four sites in Bolinao, the Philippines, and simulate future reef state for each site 40 years into the future under scenarios involving the cumulative impact of fishing, poor water quality and thermal bleaching-induced mortality related to climate change. These stressors are major threats to reef systems in Bolinao [42], [43], and the Philippines more broadly [40]. Further, both water quality and fishing pressure are suggested to be important determinants of reef resilience to climate change [10], [11], and management of both stressors is tractable, with marine reserves already being implemented extensively in the Philippines [44].

Thus our study has two distinct aims: first, to ascertain how management of local stressors might affect future trajectories of coral reefs under varying bleaching scenarios. We address this by investigating (1) the cumulative impact of fishing, poor water quality (i.e. nutrification and sedimentation) and bleaching-induced coral mortality on reef systems, and how their interactions manifest over time; and (2) patterns of reef recovery following bleaching. Our second aim is to assess the utility of simulation modelling for providing decision support for local-scale management of coral reef systems subject to multiple stressors, which involves providing guidance on which courses of management are most likely to be effective in different locations of the Bolinao reef system and over what time scales. This assessment also involves identifying the circumstances under which projections of future reef state are likely to be most certain.

## Methods

### Ethics Statement

The University of The Philippines Marine Science Institute (UPMSI) has been given Prior Informed Consent (PIC) by the Mayor of Bolinao, Pangasinan, to undertake the research from which data was drawn for this modelling analysis. The PIC is continually updated by the Deputy Director for the Bolinao Marine Laboratory. Presently the director is Dr. Ronald Villanueva, and previously, during the research cited, the director was Dr. Antonette Juinio-Menez. Since there were no materials transferred outside the country, there was no need for a gratuitous permit for materials transfer from the Bureau of Fisheries and Aquatic Resources (BFAR) of the Department of Agriculture (DA). Since the sites are not part of the National Protected Areas System, the PIC from the Mayor was sufficient. Data from 1988 & 1995 was collected by Dr. Cleto Nanola, under the UPMSI Community Ecology (ComEco) Lab with permission from Dr. Porfirio M. Aliño as the ComEco Lab director. During this period (1988 – 1995), no PIC was required to undertake field observations in non-protected areas such as those cited in the publication. For all studies only visual censuses of fish and benthic communities were conducted; no fauna or flora were collected or manipulated.

### The model

Ecological dynamics are simulated using a local-scale mean-field model developed by Fung [41] and modified by Melbourne-Thomas et al. [45] and during the course of this work. Fung et al. [41] provides a description of the model development and its general behaviours, and thus only the fundamental structure of the model is described here (also see Text S1, Figure S1, Table S1 and Table S2 for details of model equations and parameters). While Fung et al.'s [41] model uses ordinary differential equations, we represented ecological processes of shallow (∼5 – 20 m depth) coral reef systems by difference equations (see Text S1; Melbourne-Thomas et al. [45]), which simulate the biomass dynamics of three consumer functional groups (herbivorous fish, piscivorous fish and sea urchins; Table 1), and the proportional covers of five benthic functional groups (hard coral, grazed epilithic algal communities (EAC), macroturf and macroalgae; Table 1). We modified the structure of the model to include feedback between benthic structure and fish dynamics by implementing a scaling term for fish recruitment which is dependent on coral cover (based on evidence from Feary et al. [46] and Holbrook et al. [47]; see Text S2 for further details).

**Table 1:** Definition of functional groups modelled, with example taxa from the Indo-Pacific region.

Functional group	Description
Coral	Hermatypic (i.e. contain zooxanthellae) scleractinian coral species (e.g. *Pocillopora damicornis*, *Acropora* spp.).
Epilithic algal communities (EAC)	Components of the benthos such as dead coral skeletons, non-geniculate coralline algae and rocks, on which cropped filamentous algae (i.e. fine turf) of ≤∼2 – 4 mm in height grow. Fine turf exerts a constant growth pressure to form macroturf, but fine turf may be maintained by grazing of herbivorous fish and sea urchins.
Macroturf	Filamentous algal species that form turfs >∼2 – 4 mm in height (e.g. *Spyridia filamentosa*, *Hersiphonia secunda*).
Macroalgae	Brown, red and green algae which is more structurally complex and has a greater thallus size than macroturf (e.g. *Sargassum* spp., *Lobophora variegata*).
Herbivorous fish	Reef fish which feed predominantly on macroturf, macroalgae and EAC (e.g. families Siganidae, Scaridae, Acanthuridae).
Piscivorous fish	Reef associated fish which predate on herbivorous fish (e.g. families Lutjanidae, Serranidae).
Sea urchins	Grazing sea urchins which feed on EAC, macroturf and macroalgae (e.g. *Echinothrix* spp., *Diadema setosum*).

A range of natural and anthropogenic stressors can be applied as external stressor functions (hereafter ‘forcings’), that include nutrification and sedimentation (modelled as parameter scalings), and fishing and coral bleaching (which act to reduce biomass or cover; Table 2). All parameter values (both for the underlying model and external forcings) were from the literature. Where possible we used values from the literature relating to a particular functional group, otherwise we generalised the value from one or a few species across a functional group (see Fung et al. [41] and Melbourne-Thomas et al. [39] for further details).Where we had multiple values for responses to stressors, we specified these as a range, from which the model selected a value at random to introduce a stochastic element. Thus the forcing scheme represents a generalised response of coral reefs to stressors.

**Table 2:** The effect of forcings (i.e. stressors) on ecological processes and functional groups in the model.

Forcing	Implementation
Nutrification	Increases macroturf and macroalgal growth by a scaling factor of 1.3 – 2 ^(1, 2, 3)^ and 2 – 7 ^(4, 5, 6, 7, 8)^, respectively.
Sedimentation	Decreases coral growth by a scaling factor of 0 – 0.3 yr^−1(9, 10)^ and coral recruitment by 0 – 0.6 ^(11)^; increases coral mortality by 0 – 0.2 yr^−1 (12)^, and prevents recruitment of coral onto macroturf algae ^(13)^.
Coral bleaching	Coral bleaching events decreases coral cover. The number of events and the percentage of coral lost per event are specified explicitly for each scenario.
Fishing	The rate of fish extraction (t km^−2^ year^−1^) for herbivorous and piscivorous fish is specified explicitly for each scenario.

References: (1) Miller et al. [103], (2) McClanahan et al. [104], (3) McClanahan et al [105], (4) Lapointe [106], (5) Lapointe and O'Connell [107], (6) Larned [108], (7) Larned and Stimson [109], (8) Schaffelke and Klump [110], (9) Dodge et al.[111], (10) Cortes and Risk [112], (11) Babcock and Smith [113], (12) Nugues and Roberts [114], (13) Birrell et al. [115].

The model has been evaluated to ensure that it accurately captures the chief dynamic processes of coral reef systems [48], [49], such as ‘phase shifts’ from coral to algal dominance [41], which are well described for degraded coral reefs [50]. Sensitivity analysis has been undertaken for all parameters and equilibrium behaviour has been examined [51]. The model was implemented in the object-oriented Python Programming Language (Python Software Foundation, Hampton, New Hampshire, USA) version 2.5.

### Study site

**Figure 1:**
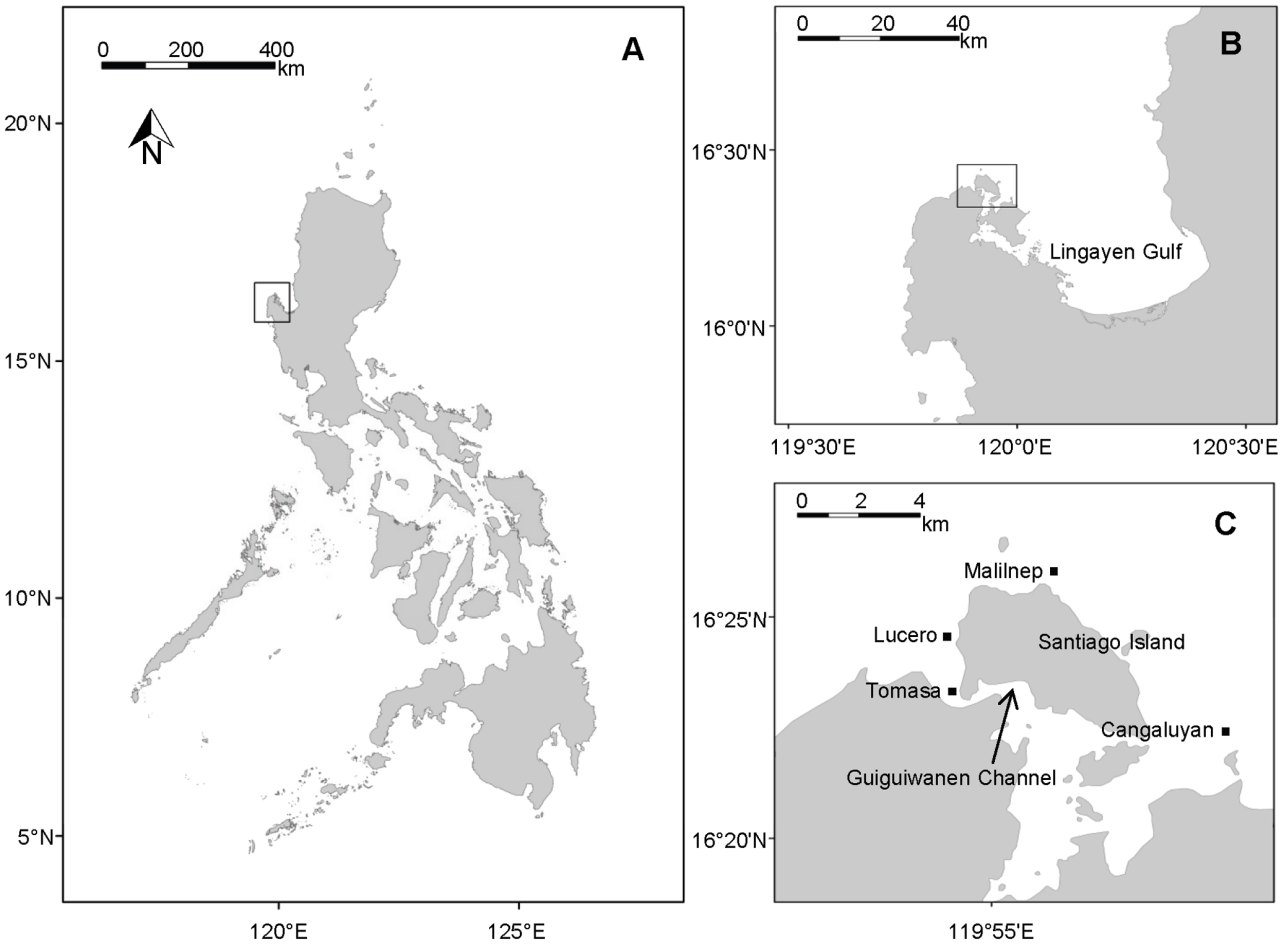
Location of study site. Location of (A) the Lingayen Gulf in the Philippines, (B) the municipality of Bolinao in the Lingayen Gulf area, and (C) the four sites (i.e. Lucero, Tomasa, Malilnep and Cangaluyan) on Bolinao's reef system for which the model was instantiated.

The model was instantiated for four sites in the coral reef system near Bolinao in the Lingayen Gulf area, the Philippines (Figure 1). Bolinao's reef system is located around Santiago Island and covers approximately 66 km^2^, including an extensive reef slope accounting for 65% of the area [52].

The Bolinao reefs are highly degraded, with the most significant stressors being poor water quality and fishing [42], [43]. Water quality has decreased significantly in Bolinao over the last two decades [53]–[55], primarily due to the development of aquaculture of milkfish (*Chanos chanos*) in the Guiguiwanen Channel since 1995 [56], [57]. The closest major river discharge is Alaminos River, located approximately 16 km to the south-east of Bolinao, but allochthonous inputs from this river are considered insignificant compared to excess fish feed and faecal matter from the fish farms, which causes nutrification and sedimentation of Boliano's reefs [56]. Artisanal fishing pressure is intense in Bolinao, with the area having one of the highest densities of fishers in the Philippines [58]. Therefore, the local fishery is highly overexploited [59]. Management of Bolinao's marine and coastal resources is the responsibility of the municipal government, as is the case in all of the Philippines since 1991 when many national government responsibilities were devolved to municipalities [60]. Existing marine management in Bolinao includes a no-take marine reserve at Lucero (one of our four sites), which was established in 2004.

The Bolinao reef system provided an ideal case study to examine local-scale management of multiple stressors because: (1) reefs are heavily affected by numerous stressors originating from anthropogenic activities and climate change; (2) marine governance is undertaken at the local-scale; and (3) data were available to parameterise and validate the model.

### Parameterisation of the model for Bolinao

The model was parameterised for four distinctly different sites on Bolinao's reef slope to capture spatial differences in local reef state, hence providing guidance on the locations where different management actions are most likely to be effective. To represent these differences we parameterised the model for four sites, *viz.* Tomasa, Lucero, Cangaluyan and Malilnep (Figure 1c). The coral reefs at Tomasa and Cangaluyan are more degraded than at Lucero and Malilnep [61].

Sea urchins are present at Lucero and Malilnep, but not at Tomasa and Cangaluyan [62], so the model was modified to exclude sea urchins for the latter two sites. The area modelled at each site was 500 m 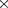 500 m because this is the scale at which marine reserves are often implemented in the area (pers. observation). The updating interval was daily. General parameter values (those not specific to each site) for the model were derived specifically for Bolinao when possible, with the remaining values derived using values measured elsewhere in the Indo-Pacific region (Table S1, Table S2). The different instantiations of the model were validated for each of the four sites following the method of Melbourne-Thomas et al. [45], i.e. we assessed whether the model could reproduce reef dynamics of the four sites from 1987 – 2008, given a known series of stressors at these sites, and data on reef state in 1987 (Figure S2, Figure S3, Figure S4, Table S3, Table S4, Text S3).

### Future management and climatic scenarios

We simulated future reef state for each of the four sites in Bolinao under 18 scenarios, which comprised of all possible combinations of different levels of fishing pressure (three levels), coral mortality due to bleaching (three levels) and water quality (two levels; Table 3).

**Table 3:** Details of the three stressors (and levels of impact) examined in future management and climatic scenarios.

Stressor	No impact	Moderate impact	High impact
Fishing pressure (F)	Reef not affected	1.3–1.4 t km^−2^year^−1^ (50% of current yield)	2.6–2.8 t km^−2^year^−1^ (current yield)
Poor water quality (i.e. nutrification and sedimentation; NS)	Reef not affected	Not modelled	Nutrification and sedimentation present (effect of forcings detailed in Table 2)
Coral bleaching (B)	Reef not affected	Reduces coral cover by 10 −60%. Occurs with a long-term frequency of once per decade	Reduces coral cover by 10 –60%. Occurs with a long-term frequency of twice per decade

We examined three scenarios of future fisheries management (Table 3). First, no fisheries management (i.e. high impact fishing pressure), with fishing pressure set at the current yield at Bolinao (i.e. 2.6–2.8 t km^−2^ year^−1^; see Text S3 for derivation). Second, a 50% reduction in fishing pressure (i.e. moderate impact fishing pressure) which could be achieved through fisheries management tools such as gear restrictions, quotas or development of alternative livelihoods for fishers. For these two scenarios fishing pressure acted on each fish functional group following Aliño et al. [63], where 68.3% of the total catch on Bolinao's reefs was herbivorous fish and 31.7% was piscivorous fish. The third scenario was implementation of no-take marine reserves which equates with zero fishing pressure (we assumed effective compliance). We do not include destructive fishing in our scenarios because fishers have ceased to employ those practices in Bolinao [64].

We represented water quality in the model through the combined effects of the nutrification and sedimentation forcings, because these two stressors co-occur in Bolinao, with both originating predominantly from aquaculture [65]. Thus management aimed at either stressor would necessarily lead to changes in the level of the other stressor. We examined two scenarios of future water quality, namely, unregulated (i.e. high impact nutrification and sedimentation) and highly regulated water quality (i.e. no impact of nutrification and sedimentation; Table 3). Unregulated water quality refers to the current situation in Bolinao, where aquaculture development is largely uncontrolled and water quality is consequently poor, leading to nutrification and sedimentation of Bolinao's reefs [66], [67]. Under the regulated water quality scenario, reefs were not subject to nutrification and sedimentation. This reduction in nutrient and sediment input could be achieved through regulation of the aquaculture industry, including restricting both the number of aquaculture cages and stocking density.

We considered climate change in terms of coral bleaching only because the effects of ocean acidification in the short- to medium-term future are uncertain [68], and the likelihood of increased frequency and/or severity of damaging typhoons due to climate change continues to be debated [69], [70]. Three scenarios for future bleaching frequency were explored, namely zero (low), one (medium) and two (high) events per decade; medium and high frequencies of bleaching were derived from Donner et al. [71] and Donner [9]. Bleaching acted to reduce coral cover by 10–60%; the upper end of this range was the average mortality of corals in Bolinao during the severe 1998 bleaching event [72], [73].

We assumed uniform magnitude of stressors for all sites. While the magnitude of bleaching, nutrification and sedimentation is likely to vary with location in the future, we did not have information on the spatial distribution of bleaching susceptibility and water quality. Further, following Fung et al. [51] and Melbourne-Thomas et al. [45], the nutrification and sedimentation forcings were based on the linear threshold model [74], and under current conditions these thresholds are exceeded for all sites (Text S3). The four instantiations of the model were parameterised to reflect reef state in each location in 2008 [61], and each scenario was modelled over 40 years for each site using Monte Carlo simulations of 20 replicate runs.

### Scenario analysis

Simulated future reef states were analysed using multivariate statistical techniques, and by examining model trajectories under these scenarios. Both approaches provide useful complementary insights into the effect of stressors on reef function. We used multivariate analyses to analyse modelled reef states for each site at four points in time selected *a priori*, *viz.* 5, 10, 20 and 40 years into the future. These are time-scales relevant to managers, and allow us to assess how interactions between stressors manifest over the long-term, including recovery patterns after bleaching. We used permutational multivariate analysis of variance (PERMANOVA) [75] to determine the significance of differences in modelled reef state, in terms of ‘location’ (i.e. mean reef state) and ‘dispersion’ (i.e. variability in reef state). The 20 model runs formed the independent replicates for the analyses.

Given that terms in the PERMANOVA can be significant due to differences in mean reef state (i.e. location) only, or differences in reef state *and* variability in reef state (i.e. dispersion), we used permutational analysis of multivariate dispersions (PERMDISP) [75] to test for homogeneity of dispersions among sets of the 20 independent model runs for each scenario. Thus, PERMDISP provided a means to: (1) clarify the nature of the significant PERMANOVA terms; and (2) quantify variability in projected trajectories within a set of Monte Carlo simulations for each scenario. This provides insights into the circumstances under which projected reef state is most certain (i.e. greater dispersion implies lower predictability). For all significant terms in the PERMANOVA we used *a posteriori* pairwise comparisons to identify the treatment groups that differed. Following the method of Anderson [76], where pairwise comparisons are undertaken, no adjustment of the significance level was made for multiple tests.

We used canonical analysis of principal coordinates (CAP) [75] and non-metric multi-dimensional scaling (MDS) [77] to visualise differences in multivariate location and dispersion, respectively, and hence to explore the nature of significant effects detected using PERMANOVA and PERMDISP. CAP is a constrained ordination technique in which between-group variability is scaled by within-group dispersion, and is thus useful to visualise results of PERMANOVA [75]. Conversely, MDS is an unconstrained ordination technique, which preserves distances between groups and thus reflects raw dispersion patterns. All multivariate analyses were based on Euclidean distances and conducted on normalised data (as consumer and benthic variables use different units, i.e. gm^−2^ and %). 4,999 permutations of the residuals under a reduced model were used to calculate *p* values for PERMANOVA, PERMDISP and CAP analyses.

We examined trajectories of reef state over 40 years for six of the 18 scenarios, representing all combinations of the different levels of fishing and water quality under the climatic scenario of decadal bleaching. These scenarios were chosen based on results from the statistical analysis and because fishing and water quality, but not bleaching, can potentially be managed at a local scale. Analysis of the trajectories allowed us to visualise changes in the functional groups driving differences in reef state detected in the PERMANOVA, and to assess whether these changes were meaningful in a management context. Further, trajectory analysis elucidates patterns of recovery following bleaching events under alternative management scenarios. Here we define coral recovery as the rate at which coral cover increases after simulated bleaching. To allow assessment of coral recovery, the magnitude and timing of bleaching events was fixed for each scenario and site for the trajectory analysis. Bleaching events were set to occur 20 and 38 years into the future and to reduce coral cover by 30% and 15% respectively.

## Results

### Interactions and impacts of multiple stressors

Modelled reef communities differed significantly under the alternative management and climatic scenarios, in terms of multivariate location (i.e. mean composition of the reef) and, in some cases, dispersion (i.e. variability of modelled communities; Table 4). There were significant interaction effects between water quality (i.e. nutrification and sedimentation, hereafter ‘nutrification-sedimentation’ or NS), fishing (F) and bleaching (B).

**Table 4:** Summary of results from PERMANOVA and PERMDISP analyses of model output under 18 scenarios of different combinations of nutrification and sedimentation (NS), fishing (F) and bleaching (B).

Site	Effect			PERMANOVA *p*(perm)					PERMDISP *p* (perm)		
				years from 2008					years from 2008		
		df	5	10	20	40	df	5	10	20	40
Malilnep	NS	1	**0.0002**	**0.0002**	**0.0002**	**0.0002**	1	**0.0002**	0.6754	**0.0003**	0.1129
	F	2	**0.0002**	**0.0002**	**0.0002**	**0.0002**	2	0.7176	**0.0077**	**0.0005**	**0.0004**
	B	2	**0.0010**	**0.0064**	**0.0002**	**0.0002**	2	0.8134	0.1817	**0.0269**	0.0875
	NS x F	2	**0.0004**	**0.0002**	**0.0002**	**0.0002**	5	**0.0002**	0.0516	0.0518	0.0632
	NS x B	2	0.0870	**0.0084**	**0.0002**	**0.0002**	5	-	0.8328	0.2008	0.2047
	F x B	4	0.0862	0.4390	0.4660	0.6748	8	-	-	-	-
	NS x F x B	4	0.0800	0.0514	0.9196	0.1606	17	-	-	-	-
Lucero	NS	1	**0.0002**	**0.0002**	**0.0002**	**0.0002**	1	**0.001**	**0.0066**	**0.0208**	**0.011**
	F	2	**0.0002**	**0.0002**	**0.0002**	**0.0002**	2	0.0946	0.0890	**0.0062**	0.7218
	B	2	**0.0004**	**0.0036**	**0.0002**	**0.0002**	2	0.8224	0.4520	**0.0002**	**0.0114**
	NS x F	2	0.3388	**0.0118**	**0.0002**	**0.0046**	5	-	**0.0176**	0.4333	0.1418
	NS x B	2	0.2612	**0.0086**	**0.0002**	**0.0008**	5	-	**0.0252**	0.3537	**0.0091**
	F x B	4	0.0740	0.3660	0.1588	0.3954	8	-	-	-	-
	NS x F x B	4	0.1108	0.6486	0.0716	0.2574	17	-	-	-	-
Cangaluyan	NS	1	**0.0002**	**0.0002**	**0.0002**	**0.0002**	1	**0.0002**	**0.0002**	**0.0002**	**0.0002**
	F	2	**0.0002**	**0.0002**	**0.0002**	**0.0002**	2	**0.0158**	0.6322	**0.0352**	0.1668
	B	2	**0.0002**	**0.0002**	**0.0002**	**0.0002**	2	**0.0194**	**0.0002**	**0.0002**	**0.0002**
	NS x F	2	**0.0002**	**0.0002**	**0.0002**	**0.0002**	5	**0.0002**	**0.0002**	**0.0002**	**0.0002**
	NS x B	2	**0.0002**	**0.0002**	**0.0002**	**0.0002**	5	**0.0002**	**0.0002**	**0.0002**	**0.0002**
	F x B	4	0.4844	**0.0062**	**0.0016**	**0.0002**	8	-	**0.0002**	**0.0002**	**0.0002**
	NS x F x B	4	0.3200	**0.0388**	**0.0160**	**0.0002**	17	-	**0.0002**	**0.0002**	**0.0002**
Tomasa	NS	1	**0.0002**	**0.0002**	**0.0002**	**0.0002**	1	0.1792	0.358	0.0582	**0.0008**
	F	2	**0.0002**	**0.0002**	**0.0002**	**0.0002**	2	**0.0002**	**0.0002**	**0.0002**	**0.0002**
	B	2	**0.0052**	**0.0002**	**0.0002**	**0.0036**	2	0.9362	0.1470	**0.0326**	0.0814
	NS x F	2	**0.0002**	**0.0002**	**0.0002**	**0.0002**	5	**0.0001**	**0.0001**	**0.0001**	**0.0001**
	NS x B	2	0.5398	**0.0002**	**0.0002**	**0.0010**	5	-	0.4491	0.1022	**0.0003**
	F x B	4	0.5980	0.0534	**0.0002**	**0.0004**	8	-	-	**0.0001**	**0.0001**
	NS x F x B	4	0.0816	0.1506	**0.0084**	**0.0008**	17	-	-	**0.0001**	**0.0001**

For each site, a PERMANOVA and a PERMDISP were conducted on simulated reef state for each of four points in time selected *a priori*, i.e. 5, 10, 20 and 40 years into the future. All analyses were conducted using normalised data, Euclidean distance and 4,999 permutations of residuals under a reduced model. Bolded values are significant at α = 0.05.

There were instances of significant interactions between all pairs of the three stressors (i.e. NS×F, NS×B, and F×B) in both the PERMANOVA and PERMDISP analyses. However, the F×B interaction occurred only where a significant three-way interaction was also evident, and thus will be analysed only in the context of the three-way interaction. In considering results, first we characterise the NS×F and NS×B interactions in terms of reef state only, through examining those terms which were significant in the PERMANOVA but did not differ in dispersion between scenarios (i.e. non-significant PERMDISP results). Second, we investigate NS×F and NS×B interactions terms which were significant in both the PERMANOVA and PERMDISP, which affords some insights into the predictability of future reef condition under alternative management and bleaching scenarios. Finally, we examine the nature of significant NS×F×B interactions, guided by our analysis of the two-way interactions.

#### Characterising two-way interactions between stressors: reef state

**Figure 2:**
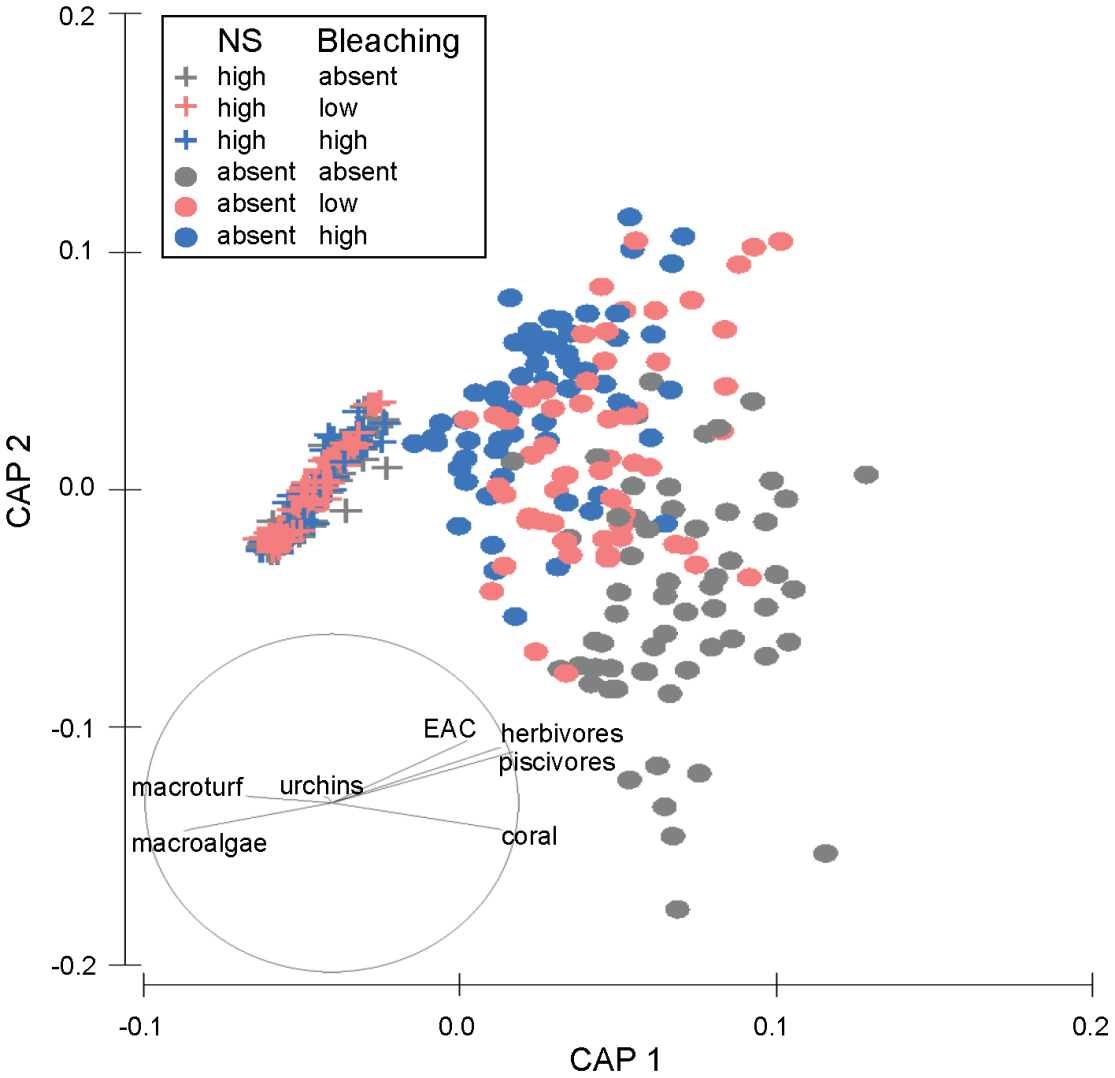
CAP ordination of modelled reef state showing the interaction between nutrification-sedimentation and bleaching. CAP ordination of modelled reef state at Lucero after 20 years indicating the nature of the two-way interaction between nutrification-sedimentation (NS) × bleaching (B), under 18 scenarios, with 20 model runs per scenario. NS had a significant effect on reef state across all levels of B (pairwise *p*<0.0001 in all cases). However, while there was a clear distinction between levels of bleaching when the disturbance acted alone (pairwise *p*<0.0007 in all cases), when it acted in conjunction with NS, there was no difference between levels of bleaching (pairwise *p*>0.1887 in all cases). A vector overlay of Spearman rank correlations (|*r*|>0.20) between functional groups and ordination axes is displayed on the plot, and indicates that modelled reef state represented by points on the left on the plot are dominated by macroturf and macroalgae, with low coral cover and fish biomass relative to reef state represented to the right hand side.

**Figure 3:**
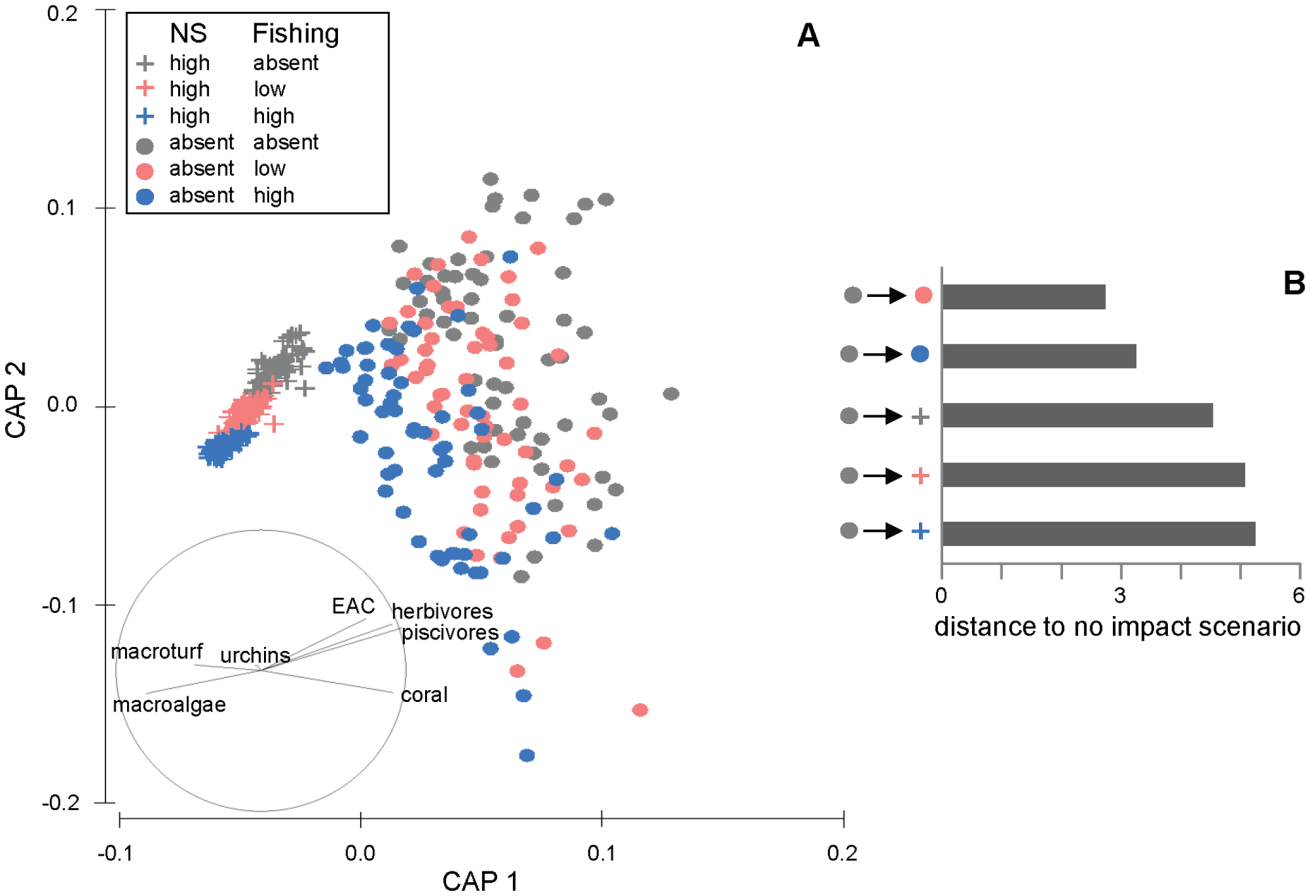
CAP ordination of modelled reef state showing the interaction between nutrification-sedimentation and fishing. CAP ordination of modelled reef state at Lucero after 20 years (a), reflecting the nature of the two-way interaction between nutrification and sedimentation (NS) × fishing (F), under 18 scenarios and 20 model runs per scenario. The average Euclidean distance between reef state under the no impact scenario and scenarios in which NS and/or fishing is present (b) indicates effect sizes. All levels of one factor were significant (pairwise *p*<0.0354 in all cases) at each level of the other factor, but the combined effect of both stressors was less than the sum of the individual effects in (b), indicating an antagonistic interaction. A vector overlay of Spearman rank correlations (|*r*|>0.20) between functional groups and ordination axes is displayed on the plot, and indicates that modelled reef state represented by points to the left on the plot are dominated by macroalgae and sea urchins, with low coral cover and fish biomass relative to reef state represented on the right hand side.

The precise nature of the NS×B and NS×F interactions differed among scenarios, but in each case were consistent across all sites and time periods. For both interactions, reef state under scenarios in which nutrification-sedimentation was present (at any level of the other stressor), was characterized by high macroalgae cover and low piscivorous and herbivorous biomass (Figure 2, 3). Given that the NS×B and NS×F interactions did not differ temporally or spatially, we present only a single example for each interaction in detail, both for Lucero after 20 years (Figure 2, 3).

For the NS×B interaction, while there was a significant distinction between levels of bleaching when this stressor acted alone, when bleaching occurred together with nutrification-sedimentation, there was no difference between the levels of bleaching (Figure 2). This was because nutrification-sedimentation was dominant to bleaching.

Conversely, for the NS×F interaction, all levels of one factor were significant at each level of the other factor, but the combined effect of both impacts was less than the sum of the individual effects (Figure 3). Following Anderson [75], we interpreted effect size as average Euclidean distance between groups. Thus the effect size of each level of NS or fishing (singularly or combined) can be assessed through looking at the average Euclidean distance between reef state under the no impact scenario and scenarios in which NS and/or fishing is present. Therefore, fishing and nutrification-sedimentation may both be described as dominant stressors; the effect of either stressor on its own degrades the reef to such an extent that there was limited scope for other stressors to cause further damage. The effect size for nutrification-sedimentation was greater than for both levels of fishing (Figure 3b), indicating that nutrification-sedimentation degraded the reef to a much greater extent than fishing. When the two impacts occurred together, poor water quality resulted in algal overgrowth of corals, with a concomitant reduction in fish biomass. In these circumstances the reef was so degraded that further loss of herbivores (i.e. grazing pressure) and piscivores due to fishing did not have a large effect on benthic composition. For NS×B and NS×F interaction terms which were significant in terms of both reef state and dispersion (i.e. significant in the PERMANOVA and PERMDISP analyses; Table 4), pairwise and CAP analyses indicated that the nature of the interactions were consistent with those described above.

#### Characterising two-way interactions between stressors: variability of modelled reef state

**Figure 4:**
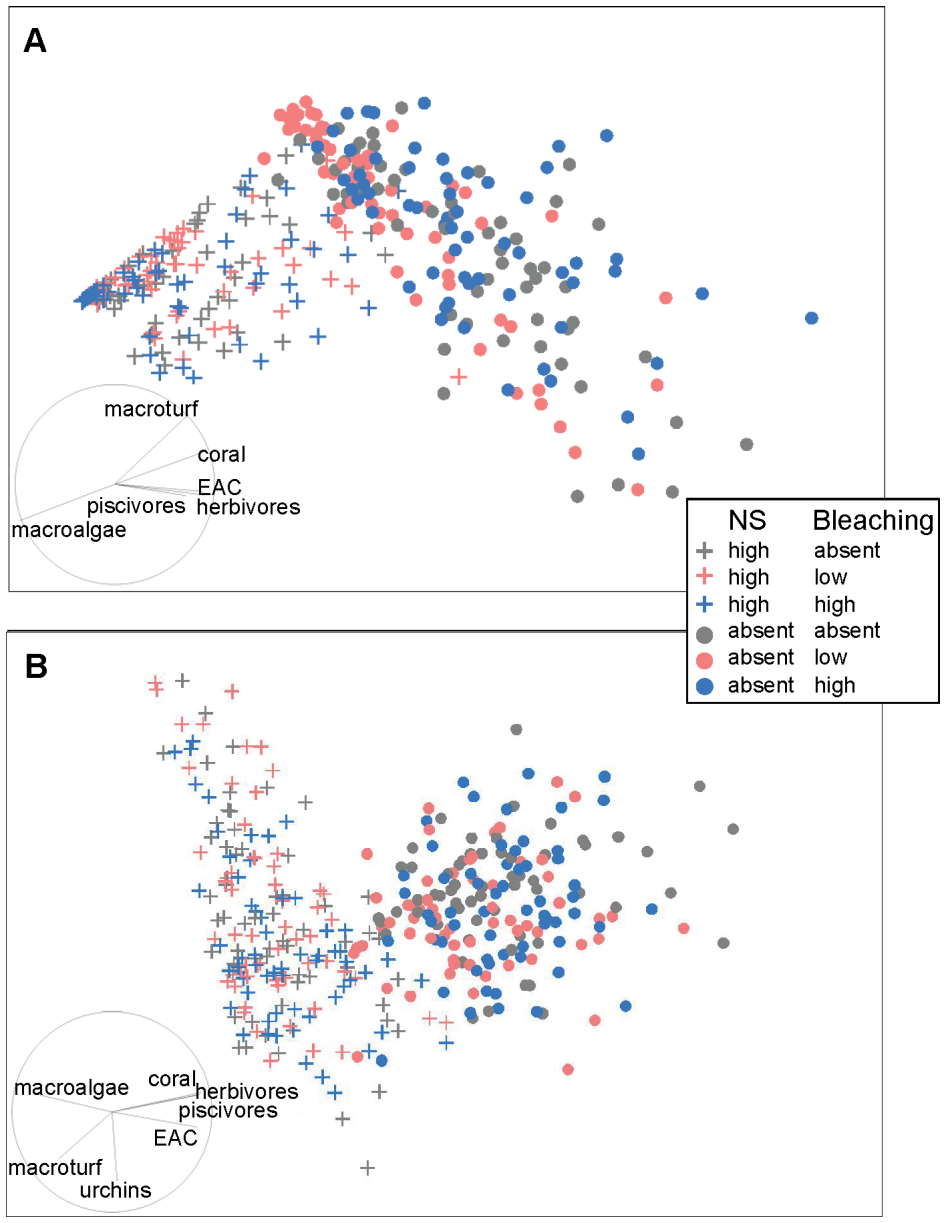
MDS ordinations of modelled reef state showing the interaction between nutrification-sedimentation and bleaching. MDS ordinations of modelled reef state showing the nature of the interaction between nutrification-sedimentation (NS) and bleaching (B) at Cangaluyan after 5 years (a) and Lucero after 10 years (b). For scenarios in which NS was not present (dots), variability in reef state increased significantly at Cangaluyan after 5 years (a; pairwise *p*<0.0018 in all cases), and was marginally significant for Lucero after 10 years (b; pairwise *p*<0.0385 in all cases). Dispersion in reef state was not significantly different between any level of B within either level of NS for either site (pairwise *p*>0.1753 in all cases). A vector overlay of Spearman rank correlations (|*r*|>0.20) between functional groups and ordination axes is displayed on the plot, and indicates that modelled reef state represented by points to the left on the plot are dominated by macroalgae, with low coral cover and fish biomass relative to reef state represented on the right hand side. 2D stress  = 0.09 (a) and 0.14 (b).

**Figure 5:**
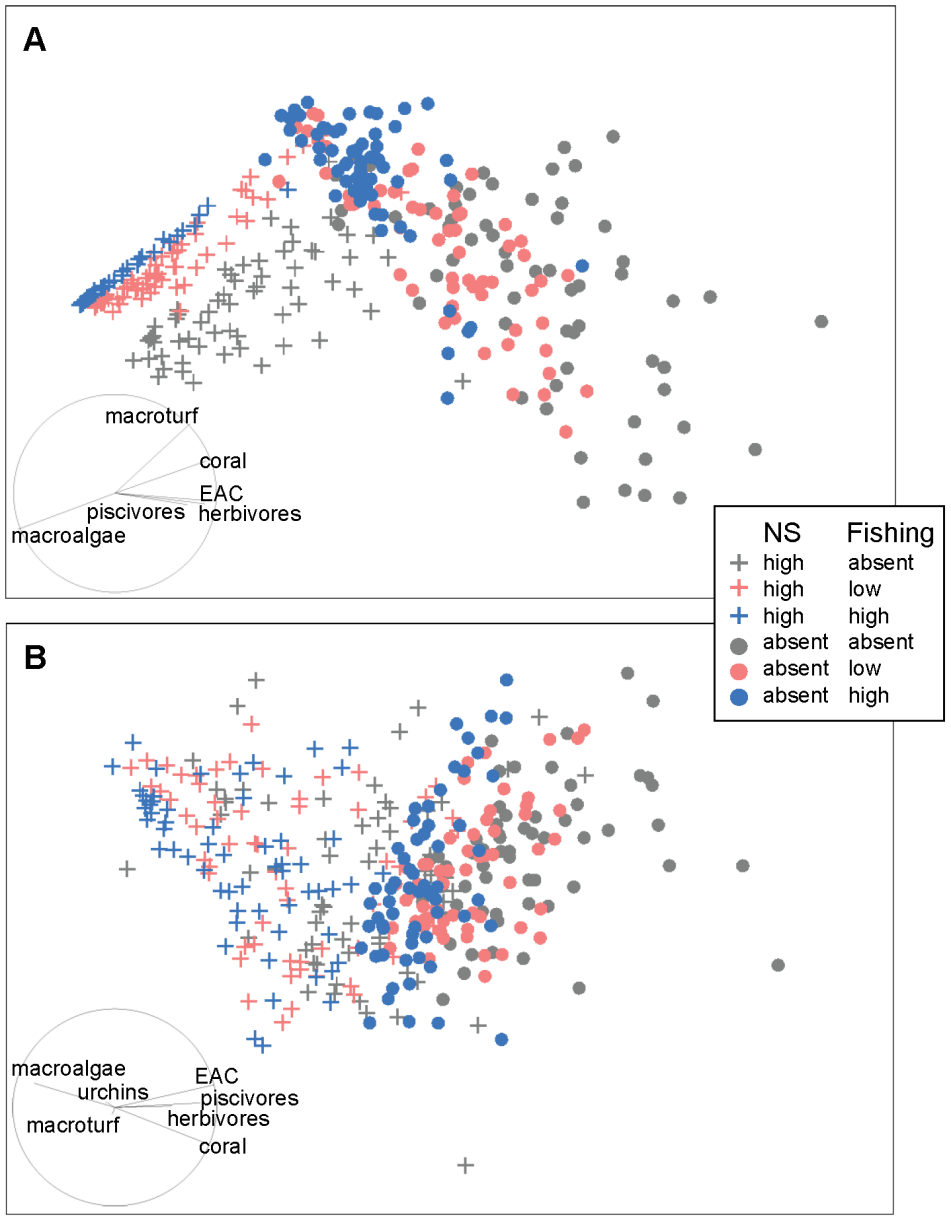
MDS ordinations of modelled reef state showing the interaction between nutrification-sedimentation and fishing. MDS ordinations of modelled reef state showing the nature of the interaction between nutrification-sedimentation (NS) and fishing (F). At Cangaluyan after 5 years (a) variability in reef state increased significantly as the magnitude of F and NS decreased (pairwise *p*<0.0385 all cases). This pattern of dispersion was also evident at Malilnep after 5 years (b) but was less clear and differences in dispersion were marginally significant (pairwise *p*<0.0463 in all cases). A vector overlay of Spearman rank correlations (|*r*|>0.20) between functional groups and ordination axes is displayed on the plot, and indicates that modelled reef state represented by points to the left on the plot are dominated by macroalgae, with low coral cover and fish biomass relative to reef state represented on the right hand side. 2D stress  = 0.09 (a) and 0.15 (b).

Patterns of dispersion differed between the NS×B and NS×F interaction, but in each case were consistent across all sites and time periods. For both kinds of interactions modelled reef state was significantly less variable for scenarios in which nutrification-sedimentation was present (Figure 4 and Figure 5), and this trend was more pronounced for the more degraded sites (i.e. Tomasa and Cangaluyan; Figure 4a and Figure 5a). Hence for both interactions we examine an example of a degraded site (Figure 4a and Figure 5a) and less degraded site (Figure 4b and Figure 5b).

For the NS×B interaction dispersion of modelled reef state did not differ significantly between levels of bleaching for either level of nutrification-sedimentation (Figure 4). However, for the NS×F interaction, variability in reef state differed significantly between levels of fishing under either level of nutrification-sedimentation, with dispersion increasing as fishing decreases (Figure 5). Again this trend was more pronounced for the more degraded sites (Figure 5a). These results suggest that future reef state is more predictable for degraded sites which are subject to high impact stressors (i.e. nutrification-sedimentation and to a less extent fishing) because these sites converge on a reef state dominated by algae with few fish and coral, and have low probability of recovery.

#### Characterising three-way interactions between stressors

Three-way interactions between nutrification-sedimentation, fishing and bleaching, were significant only for Tomasa and Cangaluyan (Table 4). All of these high order interaction terms were also significant for dispersion (i.e. significant in the PERMDISP) and therefore it is not possible to interpret the nature of the interactions solely in terms of mean reef state. However, pairwise PERMANOVA tests and PERMDISP analyses indicated that three-way interaction terms tend to manifest in the same way for both sites and all time periods. Pairwise PERMANOVA tests between each of the 18 scenarios (153 tests) indicated that differences in reef state identified for the two-way effects above were evident in the three-way interactions; namely reef state differs significantly between the different levels of fishing, bleaching, and nutrification-sedimentation, except between levels of bleaching when nutrification-sedimentation was also present. For the F×B interaction (where nutrification-sedimentation was absent) all levels of fishing were significant over all levels of bleaching. However, while all levels of bleaching were significant when fishing was not present, when fishing was present (at either level), there was no difference between scenarios in which bleaching was absent and those in which bleaching occurred at a low frequency. Note that due to the large number of *a posteriori* pairwise tests, there was a risk of compounding of Type I errors (following Anderson [76] *p* values were not adjusted to yield constant experiment-wise error rates). However, since significant terms were highly significant (*p*<0.0004 in all cases) and non-significant terms were highly non-significant (*p*>0.1259 in all cases), there is no ambiguity in the results.

Patterns in dispersion of reef state identified for the two-way interactions were similarly evident for third order interactions. Following the method of Anderson et al. [76], we plotted the mean distance-to-centroid in Euclidean distance to visualize patterns of dispersion (see Figure S5 for an example of Cangaluyan after 10 years). As we found for the two-way interactions, dispersion was lower when nutrification-sedimentation was present compared to when it was not. Within levels of nutrification-sedimentation, dispersion increased as fishing level decreased, while variability in reef state did not differ between the different levels of bleaching.

### Trajectory analysis

**Figure 6:**
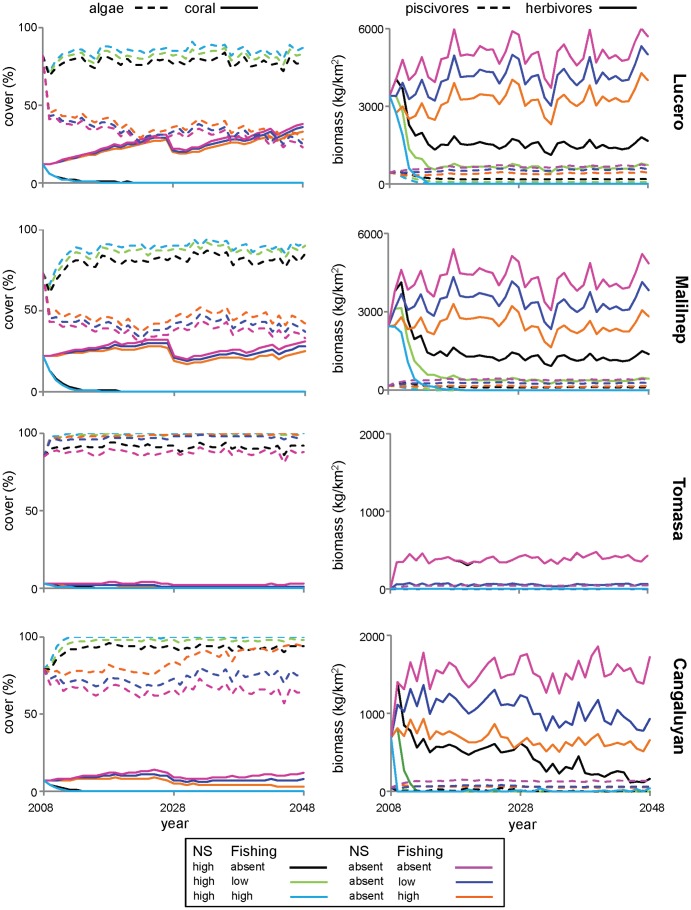
Model trajectories of benthic cover and fish biomass simulated 40 years into the future. Model trajectories of mean benthic cover and fish biomass from 20 model runs under six scenarios for all sites. The six scenarios were simulated for 40 years and were combinations of two levels of nutrification and sedimentation (NS) and three levels of fishing intensity (F) under decadal bleaching. ‘Algae’ refers to the combined covers of macroturf and macroalgal. Trajectories for sea urchins are not shown as they were stable for the entire period for all scenarios. Coral recovery after the major bleaching event in 2028 and prior to another in 2044, differed between sites and fishing scenarios.

Our analysis of reef state trajectories for various fishing and water quality scenarios under decadal bleaching revealed that differences in reef state under various scenarios detected in the PERMANOVA were driven by changes in all functional groups (Figure 6). The dominant effect of nutrification-sedimentation was evident for all sites (Figure 6). Under scenarios in which nutrification-sedimentation was absent, fish biomass and coral cover was higher, and algae cover was lower, than under scenarios in which water quality was poor. Importantly, coral cover declined to 0% within 12 years at all sites under scenarios in which reefs were subject to nutrification-sedimentation. Thus differences in reef state after 12 years under any fishing intensity (when nutrification-sedimentation was present), were driven largely by fish biomass and to a lesser extent algal cover. However, under poor water quality scenarios, the biomass of both herbivorous and piscivorous fish was less than 1400 kg km^−2^ for all sites. For reference, Nañola et al. [3] classed fish biomass at less than 5000 kg km^−2^ as ‘very low’ in their report on the status of Philippine coral reefs. Thus fish biomass was too small to be meaningful in a management context, suggesting that regulating fishing will have negligible effect when water quality is poor.

Under scenarios in which nutrification-sedimentation was not present, the effect of different levels of fishing on reef state was evident for all functional groups at all sites (except Tomasa in regards to fish). Coral cover increased as fishing pressure decreased for all sites; after 40 years coral cover was 15% higher at Lucero, 24% higher at Malilnep, 300% higher at Tomasa and 400% higher at Cangaluyan, under the zero fishing scenario compared with the high impact fishing scenario. While the differences in coral cover between fishing scenarios were highest for Tomasa and Cangaluyan, absolute coral cover was less than 3% at Tomasa and less than 12% at Cangaluyan for all scenarios. Further, at Tomasa, under all scenarios of fishing and water quality, modelled fish biomass was almost identical and was less than 500 kg km^-2^. This is largely because fish recruitment at Tomasa was constrained by low coral cover (i.e. less than 3% under all scenarios). While statistical analysis indicated that there was a significant difference between all fishing and water quality scenarios, trajectory analysis showed that these differences are not ecologically meaningful and thus are irrelevant in a management context for Tomasa. Therefore, projected responses to management were strongly non-linear and depended on the initial state of the reef.

### Coral recovery post bleaching

Analysis of the trajectories for scenarios in which nutrification-sedimentation was absent (i.e. where corals were present) highlighted that management of local stressors influenced patterns of recovery after bleaching events, although this was dependent on initial reef state (Table 5). For all sites bleaching events were modelled to occur in 2028, where coral cover was reduced by 30% (relative to cover in 2027), and in 2044, where coral cover was reduced by 15%. Loss of coral cover after each bleaching event was accompanied by an increase in algae cover and a decrease in fish biomass (which occurred because fish recruitment was impacted by reduced coral cover) for all sites. Coral recovery post-bleaching in 2028, and prior to the 2044 event, differed by site and fishing scenario. There was a clear trend in coral recovery between fishing scenarios for the least degraded sites (Lucero and Malilnep), that showed that recovery in absolute and relative terms increased as the intensity of fishing decreased (Table 5). Although this trend was apparent for Cangaluyan for scenarios in which fishing was low or absent, under high fishing pressure there was no recovery of coral cover post bleaching in 2028. This was also the case for the other highly degraded site, Tomasa, where there was no recovery under high and low fishing scenarios.

**Table 5:** Increase in coral cover between the 2028 and 2044 bleaching events for all sites under scenarios in which fishing occurred but nutrification-sedimentation was absent.

Site	Fishing scenario	Coral cover post 2028 bleaching event	Increase in coral cover between 2028 – 2044	
			absolute (% increase)	relative (% increase)
Lucero	high	20	11	55
	low	21	12	57
	absent	22	13	59
Malilnep	high	19	4	21
	low	21	5	24
	absent	22	7	32
Cangaluyan	high	5	-	-
	low	7	1	14
	absent	9	2	22
Tomasa	high	0	-	-
	low	1	-	-
	absent	2	1	50

Dashes indicate no change in coral cover.

The increase in coral cover varied between sites (in relative and absolute terms) reflecting initial reef state; i.e. changes in cover was highest at Lucero (which increased by more than 50%), followed by Malilnep and Cangaluyan. The exception to this trend was Tomasa, where the relative increase in coral cover (when fishing was present) was 50% during 2028–2044, although the absolute increase in cover was only 1%. In Tomasa and Cangaluyan during 2028–2044 changes in coral cover – i.e. very small increases in coral cover of 1–2% or maintenance of very low coral cover – were mainly due to external coral recruitment. Thus coral recovered more quickly after bleaching events at those sites which were subject to fewest additional stressors and disturbances, and were least degraded (and thus had higher endogenous recruitment). These results suggest that if coral becomes too degraded, such as at Tomasa, coral will not recover from bleaching-induced mortality irrespective of the absence of other stressors.

## Discussion

While reducing the impact of local stressors is widely advocated to bolster coral reef resilience under anticipated future changes in the climate [2], [7], the degree to which management of local stressors might affect future trajectories of reef state remains a critical knowledge gap [13]. Models are useful for understanding future reef state under alternative management and climatic scenarios, but to date have not been developed as decision support tools for local-scale management of coral reefs subject to multiple stressors. We used a simulation model of coral reefs to estimate future reef state for Bolinao, the Philippines, under alternative management and climatic scenarios. We found that management of local-scale stressors (i.e. water quality and to a lesser extent fishing) can have significant impact on future reef state. In discussing this result we first consider interactions between multiple stressors and then factors influencing coral recovery post bleaching. Next we discuss the second aim of our study, namely assessing the utility of simulation modelling as a decision support tool for management of coral reefs.

### Interactions and impacts of multiple stressors

Integral to assessing how management of local-scale stressors might influence future trajectories of reef state is investigating the cumulative impact of those stressors, including whether the effect of multiple stressors is the sum of single-stressor effects (additive), or an amplification (synergism) or a reduction (antagonism) relative to an additive effect [21]. We identified antagonistic interactions between water quality, fishing and bleaching, that were driven by a dominant stressor mechanism rather than through active opposition between the stressors. The antagonistic interaction occurred because modelled reef state was initially degraded and had limited capacity to degrade further in response to multiple stressors. For example poor water quality resulted in such a degraded reef state that there was little or no coral remaining to be bleached. The interaction identified between water quality and fishing also reflected antagonism through a dominant stressor mechanism because either stressor acted individually to degrade the reef to such an extent that there was limited scope for the other to cause further damage.

The antagonistic interactions between stressors identified in this study may be driven by the asymmetric effects of the three stressors. The individual effect of poor water quality on overall reef state was much greater than the individual effect of the two other stressors, particularly bleaching. This suggests that the interactions between poor water quality and the other two stressors are caused by the dominant impact of nutrification-sedimentation. Antagonistic interactions driven by a dominant stressor have been identified in other studies [15], [78]. In particular, Darling et al. [15] suggested that coral bleaching was driving an antagonistic interaction between bleaching and fishing, which they identified using 20 years of data from Kenyan coral reefs. Antagonistic interactions have also been identified between the effects of fishing and nutrification on macroalgae in coral reefs [24], while other studies have found this pair of stressors interact synergistically [79], [80]. This disparity of interactions identified for the same stressors highlights that interactions between stressors are likely to be dependent on context, including the initial condition of the system and the magnitude of the stressors.

Management of multiple stressors requires an understanding of how interactions between stressors manifest in different contexts. This is particularly pertinent to coral reef management given that reefs are almost always subject to more than one stressor [6], and that the nature of interactions between stressors can fundamentally affect management outcomes [19]. However, while coral reef decline has been widely attributed to the cumulative effects of multiple stressors acting simultaneously [12], [18], [81], misunderstanding of the combined impact of stressors persists [13], [23]. The effect of multiple stressors is often assumed to be the additive accumulation of impacts associated with single stressors [13], [14], and Dunne [21] asserts that in the few cases where synergies are referred to in the context of coral reefs, the term ‘synergy’ is misused. This is in part due to the inherent difficulty of teasing apart the independent and combined effects of multiple stressors [82], especially at large spatial scales. Here we demonstrate the utility of modelling for understanding the impact of cumulative stressors and suggest its application in future research on cumulative impacts.

### Coral recovery post bleaching

A critical component of understanding the importance of managing local-scale stressors to conserve coral reefs in the context of future climate change is knowledge of the factors influencing coral recovery post bleaching. We found that simulated coral recovery following bleaching was greatest at sites that were least degraded and subject to the least impact from local stressors. For the most degraded site (i.e. Tomasa), coral did not recover (under high and medium levels of fishing) or showed very little recovery (under no fishing), with a only 1% increase in absolute coral cover over 18 years. This indicates a hysteresis effect, and suggests that if coral cover becomes too depleted recovery is not possible even in the absence of stressors. This is in part because endogenous supply of coral recruits is less for degraded reefs, with a number of studies showing that coral recovery is highly dependent on the extent of remnant coral survival [83], [84]. Note that although it was beyond the scope of our study to examine the nature of phase-shifts, this model can be used to this end [41], [85].

These results support consensual understanding of conditions which foster coral resilience following bleaching-induced coral mortality [7], [8], and are consistent with experimental studies showing that reducing herbivorous fish biomass decreases coral recovery [11]. However, results from site-specific studies which assessed coral recovery inside and outside marine reserves are mixed; McClanahan [86] found no differences in recovery inside and outside reserves, while Mumby and Harborne [87] found greater recovery within marine reserves. This highlights that the extent to which marine reserves will assist coral recovery following bleaching, and other climate-induced coral mortality, will vary with context, including factors such as the presence of other concurrent stressors, coral community structure and absolute level of live coral cover post bleaching [84], [87], [88]. Indeed, in the case of Bolinao, our study suggests that if water quality is not regulated, marine reserves are unlikely to confer any significant benefits to coral health in the future.

### Managing local stressors in the face of climate change

Our research supports the paradigm that managing local-scale stressors is critical to the persistence of coral reefs in the context of global climate change, a concept that is widely advocated [2], [8] but still subject to debate [17], [18]. Our analysis of the cumulative impact of bleaching, poor water quality and fishing indicate that management of the two local stressors will significantly influence future reef state under climate change. We found that coral recovery post bleaching can be bolstered through management of fishing by building reef health prior to bleaching and aiding recovery after bleaching-induced mortality. However, the extent to which reducing fishing or improving water quality will influence future coral reef health will also vary with context [15], [87].

For our study sites at Bolinao, poor water quality emerged as the pre-eminent driver of deterioration of coral reefs, and overrode the effects of fishing and bleaching. The combined effect of excess nutrient and sediment input is well known to degrade real coral reef systems [89]–[91], and the two stressors have been suggested to interact synergistically to affect reef health [92]. For our modelled reef system the deleterious impact of poor water quality was worsened by the low level of fish grazing pressure exerted on the modelled reef, even under no fishing scenarios.

### The utility of modelling for supporting environmental management

The utility of simulation modelling to environmental management lies in the capacity of models to estimate future ecosystem state under alternative management and climatic scenarios. Through this process, models can greatly aid identification of drivers of degradation and consequently provide guidance on appropriate management actions, where they will most likely to be effective and over what time scales. Given that resources for management are limited and that management is often constrained by human use of marine resources, this information also usefully informs a ‘triage’ approach to management, whereby locations for undertaking management actions are prioritised according to where actions are most likely to be effective given existing limitations such as resources, time and knowledge.

We have demonstrated how simulation modelling can be used to support coral reef management at local scales. Our results clearly indicate that reefs in Bolinao would benefit from improving water quality above all other stressors, because even in the absence of fishing and bleaching, the present levels of nutrient enrichment and sediment deposition are likely to lead to loss of corals within a short time scale (within 12 years). Improvement in water quality could be achieved by regulating the aquaculture industry, including reducing the stocking density and number of fish cages. If water quality is improved, our results suggest that under decadal bleaching the present level of fishing can be supported at Malilnep and Lucero, and half of the present level at Cangaluyan, without further decline in coral cover. Conversely, any management intervention at Tomasa, including action to improve water quality, is unlikely to make a meaningful difference to reef health over at least a 40 year time frame. Thus, responses to management are strongly non-linear depending on the state of the reef, and management focused initially on the Lucero and Malilnep will likely yield greatest recovery for the least investment. This example highlights the importance of site-specific information in management.

Critical to using models to forecast future reef state under alternative scenarios is consideration of the predictability of those trajectories. Variability in modelled reef state (i.e. dispersion of modelled reef state between model runs) affords insights into the predictability of future reef state. We found reef state more predictable for degraded reefs, especially if the reef was subject to high impact stressors (i.e. poor water quality and to a lesser extent fishing), because these sites converge on a reef state dominated by algae with few fish and coral with low probability of recovery. Note however, that predicting the exact future state of a reef under a given scenario is not our intention, nor is it necessary to achieve good management outcomes. Rather, managers are more interested in broad-scale trends that indicate whether reefs will be broadly healthy or degraded.

Further, in considering the utility of simulation modelling to environmental management, it is important to acknowledge the host of other biological and socioeconomic factors which were not included in our modelling but which will greatly influence decision making in regards to management of fisheries, bleaching and water quality in Bolinao and elsewhere. For example, marine reserve design may be contingent upon factors such as the presence of threatened species, distribution of fishing effort by gear type or cultural practices associated with fisheries. Thus, we suggest that simulation modelling be used in combination with other decision support tools such as fisheries stock assessment models [93] or reserve zoning software [94]. Critically, simulation models, such as the one used here, are intended to provide a subset of management options, which should then be evaluated in light of the relevant socioeconomic context [39].

### Critiques and caveats

Uncertainty is inherent to modelling complex systems [95] and arises at all stages of the modelling process. When using complex systems models it is necessary to determine and describe model uncertainties, especially if the model is to be used as a decision support tool for environmental management [96]. Uncertainty in relation to model structure and function is discussed in detail in Fung et al. [41] and Melbourne-Thomas et al. [39], [45], so here we focus on uncertainties in relation to the validation of the model for Bolinao and the scenario analysis.

Uncertainty is related to our model validation process mainly due to the paucity of empirical observations of reef state in Bolinao in the past. We validated the model for each of the four sites through assessing whether the model could reproduce reef dynamics from 1987–2008 given empirical data on reef state in 1987 and a known series of stressors at these sites (Figure S2, Figure S3, Figure S4, Table S3, Table S4,Text S3). Although there was satisfactory correspondence between model trajectories and empirical data of reef state for 1987–2008, uncertainty is related to this validation process mainly because available empirical data were limited to two or three time periods for each functional group at each site. This highlights the importance of empirical data for simulation modelling of complex systems.

Given imperfect knowledge of both future impacts and how a system will react to these factors [97], scenario analysis necessarily involves simplifications and assumptions which leads to uncertainty in model projections. One of the foremost assumptions associated with predicting futures is that biological processes and relationships will not change in the future, which may not always hold [98]. For example, evidence is emerging that corals may have scope for acclimatization to predicted increases in sea surface temperature associated with climate change [99], [100]. Further, a key assumption which we made in relation to biological processes relates to recruitment of coral and fish larvae; we assumed a constant supply of external larvae. In reality, the condition of reefs close to Bolinao is likely to change in the future, which will affect exogenous recruitment to Bolinao's reefs and consequently reef state.

Uncertainty is also related to the scenarios under which projections about the future are made. For instance, the frequency of bleaching events assumed in the future scenarios modelled here were predicted using climate models [9], [71], which have their own uncertainty. Also, given uncertainty related to future frequency and nature of other climate change related stressors, such as ocean acidification and damaging storms [69], [101], we modelled climate change in terms of bleaching only. We made further simplifications given uncertainty associated with the impact of climate change on biological processes, e.g. coral growth rates remained constant in our scenarios despite some evidence that coral growth may decline under bleaching [102].

In summary, there are inevitably sources of uncertainty in scenario analysis which translates to uncertainty in the associated projections. In this sense the kind of stochastic simulation model used here is realistic because uncertainty about the future is inevitable and is captured through the model providing a probabilistic spectrum of possible futures for a given scenario. Future scenarios are intended to illustrate the range of possibilities for the future, rather than predict actual trajectories [97].

## Conclusions

Our analysis of potential reef state under alternative management and climatic scenarios highlights two key principles critical to management of coral reefs in the face of climate change. First, local-scale reef management to improve water quality and prevent overfishing can play a significant role in conserving coral reefs under the expected effects of global climate change. Second, to develop effective management actions it is critical to consider the combined impact of multiple stressors, and potential interactions between them. However, the relative importance of different stressors and how they interact is likely to vary with context, ensuring that generalization and extrapolation of specific findings is difficult. Therefore, managers require decision support tools to identify drivers of degradation for particular reefs, allowing the development of site-specific management. Simulation models, such as the one used here, are ideal tools in this endeavour, and can further support management decisions by providing insights into the efficacy of different management approaches under alternative climate (or other) scenarios. Thus we suggest that simulation modelling offers a critical and much needed opportunity for aiding local-scale management of coral reefs.

## Supporting Information

Figure S1 **The ecological processes between the seven functional groups in the local-scale mean-field model.**
(DOCX)

Figure S2 **Historical timeline of stressors to Bolinao's coral reef system during 1987 – 2008.**
(DOCX)

Figure S3 **Comparison of empirical observations and model trajectories of benthic covers in the four sites at Bolinao from 1987 – 2008.**
(DOCX)

Figure S4 **Comparison of empirical observations and model trajectories of consumer biomass in the four sites at Bolinao from 1987 – 2008.**
(DOCX)

Figure S5 **Mean distance-to-centroid in Euclidan space of the 20 predicted community states (i.e. results from Monte Carlo simulations) for each of the 18 scenarios for Cangaluyan after 10 years.**
(DOCX)

Table S1 **Parameter definitions and values derived for Bolinao, the Philippines.**
(DOCX)

Table S2 **Exogenous recruitment parameters for consumer groups used for historical trajectories.**
(DOCX)

Table S3 **Additional forcings (i.e. stressors) used in model validation.**
(DOCX)

Table S4 **Validation data for the historical reconstruction trajectories for Bolinao.**
(DOCX)

Text S1 **Model equations.**
(DOCX)

Text S2 **Additions to the model for Bolinao instantiation.**
(DOCX)

Text S3 **Model validation.**
(DOCX)
