# Supplementary material for: Modelling Coral Reef Futures to Inform Management: Can Reducing Local-Scale Stressors Conserve Reefs under Climate Change?
Source: PLoS One. 2013 Nov 18;8(11):e80137. doi: 10.1371/journal.pone.0080137 (PMC3832406; doi:10.1371/journal.pone.0080137)
Supplement: Figure S1 — The ecological processes between the seven functional groups in the local-scale mean-field model. (DOCX) [file pone.0080137.s001.docx]

Figure S1. The seven functional groups and ecological processes described by difference equations (Text S1) in the local-scale mean-field model [1]. Solid arrows represent conversion of biomass of one group to another due to ecological processes indicated by the colour of the arrow (grazing = green; growth = red; black = recruitment; blue = background mortality due to process other than fishing and predation by piscivorous fish; brown = predation; and pink = fish extraction via fishing). The direction of arrows represent an increase in cover (benthic groups) or biomass (consumer groups) of the functional group being pointed to. Arrows that do not point at a group represent loss of biomass due to exogenous factors (i.e. fishing and background mortality). The dashed black line represents competition for algae between herbivorous fish and sea urchins. The dashed orange line represents the feedback between benthic structure and fish dynamics, specifically a scaling term for fish recruitment which is dependent on coral cover (see Text S2 for further detail).

1. Fung T (2009) Local scale models of coral reef ecosystems for scenario testing and decision support. Phd thesis: University College London.
