# Supplementary material for: Modelling Coral Reef Futures to Inform Management: Can Reducing Local-Scale Stressors Conserve Reefs under Climate Change?
Source: PLoS One. 2013 Nov 18;8(11):e80137. doi: 10.1371/journal.pone.0080137 (PMC3832406; doi:10.1371/journal.pone.0080137)
Supplement: Figure S2 — Historical timeline of stressors to Bolinao's coral reef system during 1987 – 2008. (DOCX) [file pone.0080137.s002.docx]

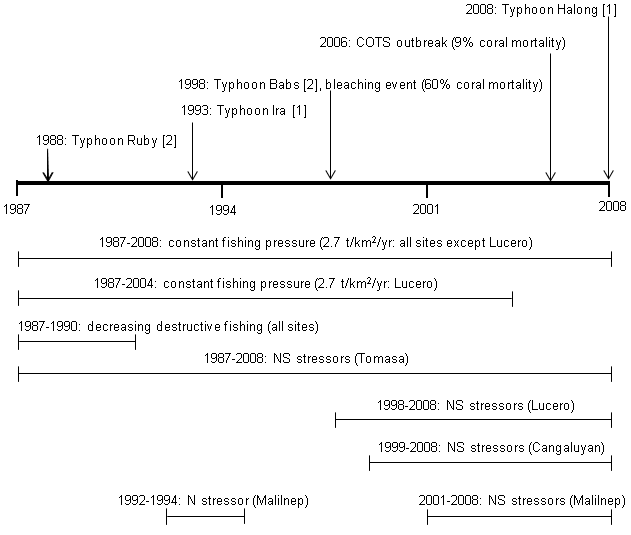


Figure S2. Historical timeline of stressors to Bolinao’s coral reef system, 1987 to 2008. Numbers in square brackets after typhoon names indicate the category of the typhoon at the time of passing within 65 km of Bolinao. ‘Destructive fishing’ refers to blast or poison fishing, where explosives or poison are used to stun fish. ‘COTS’ refers to crown-of-thorns starfish.
