# Supplementary material for: Modelling Coral Reef Futures to Inform Management: Can Reducing Local-Scale Stressors Conserve Reefs under Climate Change?
Source: PLoS One. 2013 Nov 18;8(11):e80137. doi: 10.1371/journal.pone.0080137 (PMC3832406; doi:10.1371/journal.pone.0080137)
Supplement: Figure S4 — Comparison of empirical observations and model trajectories of consumer biomass in the four sites at Bolinao from 1987 – 2008. (DOCX) [file pone.0080137.s004.docx]

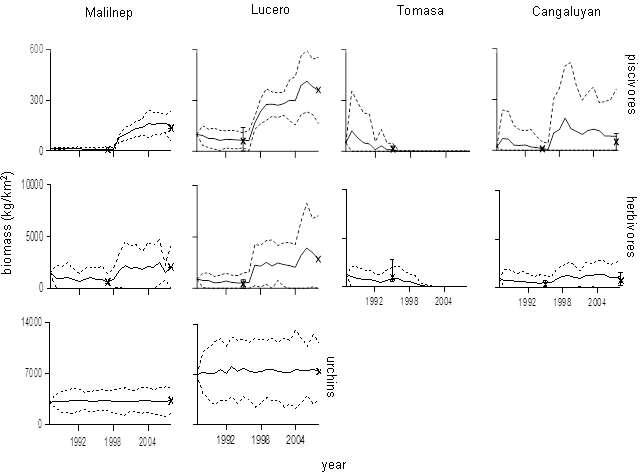


Figure S4. Comparison of empirical observations and model trajectories of consumer biomass in the four sites at Bolinao from 1987 – 2008. Solid lines represent means and dotted lines represent 95% confidence intervals from 20 model runs. Crosses and bars indicate means and ranges of observational data, respectively.
