# Supplementary material for: Modelling Coral Reef Futures to Inform Management: Can Reducing Local-Scale Stressors Conserve Reefs under Climate Change?
Source: PLoS One. 2013 Nov 18;8(11):e80137. doi: 10.1371/journal.pone.0080137 (PMC3832406; doi:10.1371/journal.pone.0080137)
Supplement: Figure S5 — Mean distance-to-centroid in Euclidan space of the 20 predicted community states (i.e. results from Monte Carlo simulations) for each of the 18 scenarios for Cangaluyan after 10 years. (DOCX) [file pone.0080137.s005.docx]

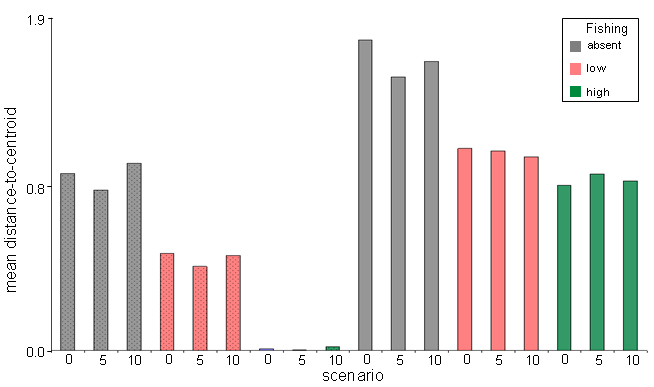


Figure S5. Mean distance-to-centroid in Euclidan space of the 20 predicted community states (i.e. results from Monte Carlo simulations) for each of the 18 scenarios for Cangaluyan after 10 years. To visualize within-group dispersion for each of the 18 scenarios, the mean distance-to-centroid for each of the 18 groups is considered; this is the mean distance in multivariate space from the centroid of the group to each of the 20 predicted community states. Scenarios in which nutrification-sedimentation is present are denoted by dotted bars, while solid bars represent scenarios in which water quality is good. The numbers along the scenario axis refer to different bleaching scenarios; ‘0’ is no bleaching, ‘5’ is bleaching every 5 years and ‘10’ is bleaching every 10 years. The colour of the bars refers to the fishing intensity. Variability in predicted communities is greater in scenarios in which nutrification is not present, and within levels of nutrification-sedimentation dispersion increases as fishing intensity decreases. There is little difference in dispersion between bleaching scenarios within nutrification-sedimentation and fishing scenarios.
