# Supplementary material for: Modelling Coral Reef Futures to Inform Management: Can Reducing Local-Scale Stressors Conserve Reefs under Climate Change?
Source: PLoS One. 2013 Nov 18;8(11):e80137. doi: 10.1371/journal.pone.0080137 (PMC3832406; doi:10.1371/journal.pone.0080137)
Supplement: Table S1 — Parameter definitions and values derived for Bolinao, the Philippines. (DOCX) [file pone.0080137.s006.docx]

Table S1. Parameter definitions and values derived for Bolinao, the Philippines. Values were derived by Fung et al. [1,2], except for the term for fishing recruitment which is dependent on coral cover (further detail in Text S2). Benthic functional groups are represented by proportional cover and consumer groups by biomass (kg/km^2^). Parameter restrictions stated in the footnotes are necessary to keep state variables within realistic biological ranges, i.e. benthic covers ≥ 0 and ≤ 1, and consumer biomasses ≥ 0 (but without tending to infinity). See Fung et al. [1,2] for details on mathematical derivations of parameter restrictions.

| Parameter | Definition | | Derived value(s) | |
| --- | --- | --- | --- | --- |
| $l_{C}^{ex}$ | The rate of exogenous coral recruitment. | | 0.00004 – 0.01 yr^-1^ | |
| $l_{C}^{en}$ | The rate of endogenous coral recruitment. | | 0.0009 – 0.41 yr^-1^ | |
| $\varepsilon_{C}$ | Coefficient for the recruitment rate of coral onto macroturf, relative to the rate onto grazed EAC. | | 0.05 – 0.15 | |
| $r_{C}$ | The growth rate of existing coral over grazed EAC. | | 0.1 – 0.2 yr^-1^ | |
| $\beta_{M}$ | Coral growth is inhibited by the presence of nearby macroalgae and this is represented as depression of $r_{C}$ by the factor$(1-\beta_{M}M)$. | | 0.2 – 0.3 yr^-1^ | |
| $\alpha_{C}$ | Coefficient for the growth rate of coral over macroturf, relative to its growth over grazed EAC. | | 0 – 1 | |
| $d_{C}$ | The background mortality rate of corals. | | 0.02 – 0.19 yr^-1^ | |
| $\gamma_{MC}$ | Coefficient for the growth rate of macroalgae over coral, relative to its growth over grazed EAC. | | 0 – 0.9 | |
| $r_{M}$ | The growth rate of macroalgae over grazed EAC. | | 0.2 – 1.15 yr^-1^ | |
| $\zeta_{T}$ | The rate at which grazed EAC grows onto macroturf. | | 2 – 20 yr^-1^ | |
| $g_{T}$ | The maximum rate (per unit of grazing pressure$\theta$) at which existing macroturf is grazed down. | | 5 – 15 yr^-1^ | |
| $\gamma_{MT}$ | Coefficient for the growth rate of macroalgae over macroturf, relative to its growth over grazed EAC. | | 0 – 0.9 | |
| $g_{M}$ | The maximum rate (per unit of grazing pressure $\theta$) at which existing macroalgae is grazed down. | | 0.01$g_{T}$ – $g_{T}$yr^-1^ | |
| $i_{H}$^(1)^ | A parameter that measures the inaccessibility of algae (macroturf and macroalgae) to herbivorous fish grazing. | | $i_{H\min}$– 1 × 10^4^ kg km^-2^ | |
| $i_{U}$^(2)^ | A parameter that measures the inaccessibility of algae (macroturf and macroalgae) to urchin grazing. | | $i_{U\min}$ – 3 × 10^3^ kg km^-2^ | |
| $l_{H}^{ex}$ | The rate of exogenous recruitment of herbivorous fish. | | See Table S2 | |
| *continued on next page* | | | | |
| *continued from previous page* | | | | |
| $l_{H}^{en}$ | | Coefficient for the rate of endogenous recruitment of herbivorous fish. | | 0 – 0.3 yr^-1^ |
| $\lambda_{U}$ | | A parameter that determines the competitiveness of urchins relative to herbivorous fish. | | 0.2 |
| $\delta$ | | A scaling factor to fish recruitment which measures how survival of recruits changes with coral cover. | | 0.25 where *C* < 5%,  *aC*/[1 + (*a/b)C^d^*] where  5 % ≤ *C* ≤ 60%,  1 where *C* > 60% ^(3)^ |
| $\mu_{M},\mu_{T},\mu_{E}$^(4)^ | | The herbivorous fish biomass accumulated from grazing on 100% cover of macroalgae, macroturf and EAC respectively, and which contributes to somatic growth of herbivorous fish. | | 9 × 10^2^ –$\mu_{\max}$kg km^-2^ yr^-1^ |
| $d_{H}$^(5)^ | | The mortality rate of herbivorous fish from all factors other than predation by piscivorous fish and fishing. | | $d_{H\min}$ – 4 yr^-1^ |
| $g_{P}$ | | The maximum predation rate of piscivorous fish on herbivorous fish. | | 0.9 – $g_{P \max}$ yr^-1^ |
| $i_{PH}$ | | A parameter that measures the inaccessibility of herbivorous fish to predation by piscivorous fish. | | 7 × 10^3^ – 1 × 10^4^ kg km^-2^ |
| $\rho_{H}$ | | The proportion of the total fishing pressure which acts on herbivorous fish. | | 0 – 1,  where $\rho_{H}+\rho_{P}=1$ |
| $f$ | | The maximum fish biomass that can be caught through fishing (i.e. the fishing forcing). | | Parameterized by the model user |
| $i_{FH}$ | | A parameter that measures the inaccessibility of herbivorous fish to fishermen. | | 0 – 0.0001 kg km^-2^ |
| $l_{P}^{ex}$ | | The rate of exogenous recruitment of piscivorous fish. | | See Table S2 |
| $l_{P}^{en}$ | | Coefficient for the rate of endogenous recruitment of piscivorous fish. | | 0 – 0.1 yr^-1^ |
| $r_{P}$ | | The proportion of consumed biomass which is used for somatic growth, for piscivorous fish. | | 0.03 – 0.2 |
| $d_{P}$^(6)^ | | The mortality rate of piscivorous fish from all factors other than predation and fishing. | | $d_{P\min}$ – 1 yr^-1^ |
| $\psi_{P}$ | | Coefficient for the predation rate on piscivorous fish by piscivorous fish, relative to that on herbivorous fish. | | 0.01 – 7 |
| $i_{PP}$ | | A parameter that measures the inaccessibility of piscivorous fish to predation by other piscivorous fish. | | 7 × 10^3^ – 1 × 10^4^ kg km^-2^ |
|  | |  | | *continued on next page* |
| *continued from previous page* | | | |  |
| $\rho_{P}$ | | The proportion of the total fishing pressure which acts on piscivorous fish. | | 0 – 1,  where $\rho_{H}+\rho_{P}=1$ |
| $i_{FP}$ | | A parameter that measures the inaccessibility of piscivorous fish to fishermen. | | 0 – 0.0001 kg km^-2^ |
| $l_{U}^{ex}$ | | The rate of exogenous recruitment of sea urchins. | | See Table S2 |
| $\kappa_{U}$ | | A parameter that measures the biomass accumulated by urchin grazing and which contributes to somatic growth relative to that for herbivorous fish grazing. | | 0.7 – 3 |
| $l_{U}^{en}$ | | Coefficient for the rate of endogenous recruitment of sea urchins. | | 0 – 0.002 yr^-1^ |
| $\lambda_{H}$ | | A parameter that determines the competitiveness of herbivorous fish relative to urchins. | | 0.8,  where 1 – $\lambda_{U}$ |
| $d_{U}$^(7)^ | | The mortality rate of urchins. | | $d_{U\min}$– 0.6 yr^-1^ |

(1) $i_{H\min}=\max\left\{1000,\left|\frac{900\max\left\{g_{T},\zeta_{T}\right\}}{4-0.000001}\right|\right\}$

(2) $i_{U\min}=\max\left\{2000,\left|\frac{900\kappa_{U}\max\left\{g_{T},\zeta_{T}\right\}}{0.6-0.000001}\right|\right\}$

^(3)^ Holbrook et al. [3] and Feary et al. [4]. Where *C* is coral cover, and *a, b* and *d* are fitted parameters. We used *a* =101, *b* = 1.1 and *d* = 0.83. See Text S2.

^(4)^ $\mu_{\max}=\min\left\{500000,\mu_{\max1}\right\}$, where $\mu_{\max1}=\min\left\{\frac{i_{H}\left(4-0.000001\right)}{\max\left\{g_{T},\zeta_{T}\right\}},\frac{i_{U}\left(0.6-0.000001\right)}{\kappa_{U}\max\left\{g_{T},\zeta_{T}\right\}}\right\}$

^(5)^ $d_{H\min}=\max\left\{0.4,d_{H\min1}\right\}$, where

$d_{H\min1}=\max\left\{\left|\frac{\mu\max\left\{g_{T},\zeta_{T}\right\}}{i_{H}}\right|+0.000001,\left|l_{H}^{en}\right|+0.000001\right\}$ with $\mu_{M}=\mu_{T}=\mu_{E}=\mu$

(6) $d_{P\min}=\max\left\{0.15,\left| l_{P}^{en}+r_{P}g_{P}\right|+0.000001\right\}$ (7) $d_{U\min}=\max\left\{0.05,\left|\frac{\kappa_{U}\mu\max\left\{g_{T},\zeta_{T}\right\}}{i_{U}}\right|+0.000001\right\}$

**REFERENCES**

1. Fung T (2009) Local scale models of coral reef ecosystems for scenario testing and decision support. Phd thesis: University College London.

2. Fung T, Seymour RM, Johnson CR (2011) Alternative stable states and phase shifts in coral

reefs under anthropogenic stress. Ecology 92: 967-982.

3. Holbrook SJ, Schmitt RJ, Brooks AJ (2008) Resistance and resilience of a coral reef fish community to changes in coral cover. Marine Ecology Progress Series 371: 263-271.

4. Feary D, Almany G, McCormick MI, Jones GB (2007) Habitat choice, recruitment and the response of coral reef fishes to coral degradation. Oecologia 153: 727-737.
