# Supplementary material for: Modelling Coral Reef Futures to Inform Management: Can Reducing Local-Scale Stressors Conserve Reefs under Climate Change?
Source: PLoS One. 2013 Nov 18;8(11):e80137. doi: 10.1371/journal.pone.0080137 (PMC3832406; doi:10.1371/journal.pone.0080137)
Supplement: Table S2 — Exogenous recruitment parameters for consumer groups used for historical trajectories. (DOCX) [file pone.0080137.s007.docx]

Table S2. Exogenous recruitment parameters for consumer groups used for historical trajectories. Exogenous recruitment of herbivorous fish, pisivorous fish and urchins are denoted by $l_{H}^{ex}$, $l_{P}^{ex}$ and $l_{U}^{ex}$, respectively. Rates of recruitment were estimated from McManus et al. [1] and were refined through model tuning.

| **Site** | **Exogenous recruitment parameters** | | |
| --- | --- | --- | --- |
|  | $l_{H}^{ex}$ (kg km^-2^yr^-1^) | $l_{P}^{ex}$ (kg km^-2^yr^-1^) | $l_{U}^{ex}$ (kg km^-2^yr^-1^) |
|
| Tomasa | 1.30 × 10^3^ –  4.30 × 10^3^ | 8.00 × 10^2^ –  1.83 × 10^3^ | - |
| Cangaluyan | 1.30 × 10^3^ –  5.29 × 10^3^ | 9.00 × 10^2^ –  2.24 × 10^3^ | - |
| Malilnep | 3.20 × 10^3^ –  8.54 × 10^3^ | 1.45 × 10^2^ –  2.70 × 10^3^ | 0 –  1.96 × 10^3^ |
| Lucero | 2.50 × 10^3^ –  1.03 × 10^4^ | 1.40 × 10^2^ –  5.07 × 10^3^ | 0 –  4.23 × 10^3^ |

**REFERENCES**

1. McManus J, Nanola C, Reyes R, Kesner K (1992) Resource ecology of the Bolinao coral reef system. Manila, the Philippines: International Center for Living Aquatic Resources Management (ICLARM) Studies and Reviews 22.
