# Supplementary material for: Modelling Coral Reef Futures to Inform Management: Can Reducing Local-Scale Stressors Conserve Reefs under Climate Change?
Source: PLoS One. 2013 Nov 18;8(11):e80137. doi: 10.1371/journal.pone.0080137 (PMC3832406; doi:10.1371/journal.pone.0080137)
Supplement: Table S3 — Additional forcings (i.e. stressors) used in model validation. (DOCX) [file pone.0080137.s008.docx]

Table S3. Additional forcings (i.e. stressors) used in model validation. Effect on ecological processes and functional groups in the model.

| **Forcing** | **Implementation** |
| --- | --- |
| Typhoons | Typhoons (i.e. wind speeds ≥ 118 km hr^-1^ ^(1)^) decrease coral cover by 0.2 – 20% ^(2,3)^ and macroalgal cover by 45 – 90% ^(4)^. |
| Blast fishing | Decreases coral cover by 0.14% yr^-1^ and increases fishing extraction by 1% yr^-1^ at a blasting rate of 1 blast hr^-1(5,6,7)^. Damage inflicted by blast fishing is assumed to increase linearly with blasting rate. Blasting rate is specified for each scenario. |
| Poison fishing | Decreases coral cover by 0.01% yr^-1^ poison-fisher^-1 (5)^. Damage inflicted by poison fishing is assumed to increase linearly with the number of poison fishers. Number of poison fishers is specified for each scenario. |

References: (1) Guard and Lander [1], (2) Cheal et al. [2], (3) Gardner et al. [3], (4) Mumby et al. [4], (5) McManus et al. [5], (6) McManus et al. [6], (7) Calud et al. [7].

**REFERENCES**

1. Guard C, Lander M (1999) A scale relating tropical cyclone wind speed to potential damage for the Pacific Ocean region: A user's manual. Mangilao, USA: Water and Environmental Research Institute, University of Guam.

2. Cheal AJ, Coleman G, Delean S, Miller I, Osborne K, et al. (2002) Responses of coral and fish assemblages to a severe but short-lived tropical cyclone on the Great Barrier Reef, Australia. Coral Reefs 21: 131-142.

3. Gardner TA, Cote IM, Gill JA, Grant A, Watkinson AR (2005) Hurricanes and Caribbean coral reefs: Impacts, recovery patterns, and role in long-term decline. Ecology 86: 174-184.

4. Mumby PJ, Hedley JD, Zychaluk K, Harborne AR, Blackwell PG (2006) Revisiting the catastrophic die-off of the urchin *Diadema antillarum* on Caribbean coral reefs: Fresh insights on resilience from a simulation model. Ecological Modelling 196: 131-148.

5. McManus J, Reyes R, Nanola C (1997) Effects of some destructive fishing methods on coral cover and potential rates of recovery. Environmental Management 21: 69-78.

6. McManus J, Nanola C, Reyes R, Kesner K (1992) Resource ecology of the Bolinao coral reef system. Manila, the Philippines: International Center for Living Aquatic Resources Management (ICLARM) Studies and Reviews 22.

7. Calud A, Rodriguez J, Aruelo R, Aguilar G, Cinco E, et al. Preliminary results of a study of the municipal fisheries in Lingayen Gulf. In: Silvestre G, Miclat E, Chua T, editors; 1989; LaUnion. pp. 3-18.
