# Supplementary material for: Modelling Coral Reef Futures to Inform Management: Can Reducing Local-Scale Stressors Conserve Reefs under Climate Change?
Source: PLoS One. 2013 Nov 18;8(11):e80137. doi: 10.1371/journal.pone.0080137 (PMC3832406; doi:10.1371/journal.pone.0080137)
Supplement: Table S4 — Validation data for the historical reconstruction trajectories for Bolinao. (DOCX) [file pone.0080137.s009.docx]

Table S4. Validation data for the historical reconstruction trajectories for Bolinao.

| Variable | Site | Year | Source | Mean | | Range |
| --- | --- | --- | --- | --- | --- | --- |
| coral cover (%) | Tomasa | 1988 | a | | 52.7 | - |
|  |  | 1995 | a | | 21.3 | - |
|  |  | 2007 | b | | 2.7 | - |
|  | Cangaluyan | 1988 | c | | 55.3 | - |
|  |  | 1995 | a | | 22.1 | - |
|  |  | 2008 | b | | 7.1 | 6.3 – 9.1 |
|  | Lucero | 1988 | c | | 64.1 | - |
|  |  | 1995 | a | | 28.7 | - |
|  |  | 2008 | b | | 11.9 | 0.9 – 22.8 |
|  | Malilnep | 1988 | c,d | | 53.4 | 51.1 – 55.4 |
|  |  | 1997 | e | | 43.5 | - |
|  |  | 2006 | b | | 17.3 | 0.1 – 26.4 |
|  |  | 2008 | b | | 22.0 | - |
| macroturf algae | Tomasa | 1988 | a | | 32.0 | - |
| cover (%) |  | 1995 | a | | 50.9 | - |
|  |  | 2007 | b | | 71.2 | - |
|  | Cangaluyan | 1988 | c | | 6.1 | - |
|  |  | 1995 | a | | 40.4 | - |
|  |  | 2008 | b | | 66.1 | 50.1 – 79.1 |
|  | Lucero | 1988 | c | | 11.3 | - |
|  |  | 1995 | a | | 58.2 | - |
|  |  | 2008 | b | | 62.7 | 61.7 – 36.7 |
|  | Malilnep | 1988 | c,d | | 8.5 | 7.3 – 8.9 |
|  |  | 1997 | e | | 40.8 | - |
|  |  | 2006 | b | | 70.9 | 58.3 – 89.2 |
|  |  | 2008 | b | | 63.5 | - |
| macroalgae | Tomasa | 1988 | a | | 4.8 | - |
| cover (%) |  | 1995 | a | | 1.7 | - |
|  |  | 2007 | b | | 13.7 | - |
|  | Cangaluyan | 1988 | c | | 1.2 | - |
|  |  | 1995 | a | | 18.3 | - |
|  |  | 2008 | b | | 12.6 | 7.4 – 23.4 |
|  | Lucero | 1988 | c | | 0.5 | - |
|  |  | 1995 | a | | 0.4 | - |
|  |  | 2008 | b | | 18.6 | 8.5 – 28.7 |
|  | Malilnep | 1988 | c,d | | 1.6 | - |
|  |  | 1997 | e | | 2.5 | - |
|  |  | 2006 | b | | 5.7 | 0.9 – 9.9 |
|  |  | 2008 | b | | 10.4 | - |
| herbivorous fish | Tomasa | 1988 | - | | - | - |
| biomass (kg/km^2^) |  | 1995 | a | | 98.0 | 53.0 – 276.0 |
|  |  | 2007 | - | | - | - |
|  | Cangaluyan | 1988 | c | | 230.0 | - |
|  |  | 1995 | a | | 320.0 | 69.0 – 700.0 |
| *continued from previous page* | | | | | | |
|  |  | 2008 | a | | 690.0 | 240.0 – 1510.0 |
|  | Lucero | 1988 | c | | 230.0 | - |
|  |  | 1995 | a | | 429.0 | 69.0 – 840.0 |
|  |  | 2008 | a | | 2840.0. | - |
|  | Malilnep | 1988 | c | | 399.0 | - |
|  |  | 1997 | f | | 600.0 | 510.0 – 640.0 |
|  |  | 2008 | f | | 2036.0 | - |
| piscivorous fish | Tomasa | 1988 | - | | - | - |
| biomass (kg/km^2^) |  | 1995 | a | | 13.5 | 6.7 – 31.0 |
|  |  | 2007 | - | | - | - |
|  | Cangaluyan | 1988 | c | | 87.0 | - |
|  |  | 1995 | a | | 9.01 | 2.0 – 15.0 |
|  |  | 2008 | a | | 5.0 | 0.0 – 10.0 |
|  | Lucero | 1988 | c | | 100.0 | - |
|  |  | 1995 | a | | 61.0 | 2.0 – 140.0 |
|  |  | 2008 | a | | 362.0 | - |
|  | Malilnep | 1988 | c | | 20.0. | - |
|  |  | 1997 | f,g | | 7.6 | 5.3 – 10.0 |
|  |  | 2008 | f | | 14.0 | - |
| sea urchin | Lucero | 2007 | h | | 7370.0 | - |
| biomass (kg/km^2^) | Malilnep | 2007 | h | | 3250.0 | - |

Sources: (a) UPMSI (unpublished data), (b) Vergara [1], (c) Menez et al. [2],(d) PhilReefs [3], (e) Cesar et al. [4], (f) Geronimo [5], (g) Pet-Soede [6], (h) Geronimo [5].

**REFERENCES**

1. Vergara MWR (2009) Coral community structure of the Bolinao Reef System, Northwestern Philippines. MSc Thesis: University of the Philippines, Manila.

2. Menez L, McManus L, Metra N, Jimenez J, Rivera C, et al. Survey of the coral resource of western Lingayen Gulf, Philippines; 1991; Manila. pp. 77-82.

3. PhilReefs (2003) Reefs through Time 2003: Workshop Proceedings. Quezon City and the Marine Parks Centre, Tokyo: Coral Reef Information Network of the Philippines (PhilReefs), University of the Philippines Marine Science Institute. 197 p.

4. Cesar H, Pet-Soede L, Quibilan MCC, Alino P, Arceo H, et al. (2001) First evaluation of the impacts of the 1998 coral bleaching event to fisheries and tourism in the Philippines. In: Schuttenberg H, editor. Coral bleaching: Causes, consequences and responses (Selected papers presented at the 9th International Coral Reef Symposium, October 2000). Rhode Island, USA: Coastal Resources Center.

5. Geronimo RC (2009) Trophic dynamics of the Santiago Island Reef Slope (Pangasinan, Philippines). MSc Thesis: University of the Philippines, Manila.

6. Pet-Soede L (2000) Effects of coral bleaching on the socio-economics of the fishery in Bolinao, Pangasinan, Philippines. MSc thesis. Manila: University of the Philippines.
