# Supplementary material for: Modelling Coral Reef Futures to Inform Management: Can Reducing Local-Scale Stressors Conserve Reefs under Climate Change?
Source: PLoS One. 2013 Nov 18;8(11):e80137. doi: 10.1371/journal.pone.0080137 (PMC3832406; doi:10.1371/journal.pone.0080137)
Supplement: Text S1 — Model equations. (DOCX) [file pone.0080137.s010.docx]

**SUPPLEMENTARY MATERIAL**

**Text S1: Model equations**

Ecological dynamics in the local-scale mean-field model developed by Fung [1], also see Fung et al. [2], and modified by Melbourne-Thomas et al. [3], are defined by seven difference equations (A1 – A2 and A6 – A9), which can be parameterized for different bio-geographic areas. Parameter definitions and values derived for Bolinao, the Philippines, are provided in Table S1 and S2. The seven functional groups (state variables) modeled are hard coral (*C*), macrotruf algae (*T*), macroalgae (*M*), epilithic algal communities or EAC (*E*; where *E* = 1 – *C* – *T* – *M*), herbivorous fish (*H*), piscivorous fish (*P*) and urchins (*U*). Benthic groups (coral and algae) are quantified by proportional covers and the consumer groups (fish and urchins), by biomass.

At time *t +* 1 the proportional cover of hard coral ($C'$) is described by the following equation

**$C'=C+\left(l_{C}^{ex}+l_{C}^{en}C\right)(E+\varepsilon_{C}T)+r_{C}(1-\beta_{M}M)(E+\alpha_{C}T)C-d_{C}C-\gamma_{MC}r_{M}MC$,** (A1)

and the proportional cover for macroturf algae ($T'$) is given by

$T'=T+\zeta_{T}(1-\theta)E-g_{T}\theta T-\varepsilon_{C}(_{C}^{s}+l_{C}^{b}C)T-r_{C}\alpha_{C}(1-\beta_{M}M)CT-\gamma_{MT}r_{M}MT$, (A2)

where *θ* represents grazing pressure and is described by

$\theta=\theta_{H}+\theta_{U}$, (A3)

$\theta_{H}=\left(\frac{H}{i_{H}+H}\right)$, (A4)

$\theta_{U}=\left(\frac{U}{i_{U}+U}\right)$. (A5)

The proportional cover of macroalgae ($M'$) at time *t* + 1 is

$M'=M+r_{M}ME-g_{M}\theta M+\gamma_{MC}r_{M}MC+\gamma_{MT}r_{M}MT$ (A6)

At time *t* + 1 the biomass of herbivorous fish ($H'$) is given by

$$H'=H+\delta\left(l_{H}^{ex}+l_{H}^{en}H\right)+\theta_{H}(1-\lambda_{U}\theta_{U})\left(g_{M}M\mu_{M}+g_{T}T\mu_{T}+\zeta_{T}E\mu_{E}\right)-d_{H}H-g_{P}\left(\frac{H^{2}}{i_{PH}^{2}+H^{2}}\right)$$

$-\rho_{H}f\left(\frac{H}{i_{FH}+H}\right)$, (A7)

and the biomass of piscivorous fish is

$$P'=P+\delta\left(l_{P}^{ex}+l_{P}^{en}P\right)+r_{P}g_{P}\left(\frac{H^{2}}{i_{PH}^{2}+H^{2}}\right)P-d_{P}P-\psi_{P}g_{P}(1-r_{P})\left(\frac{P^{2}}{i_{PP}^{2}+P^{2}}\right)P$$

$-\rho_{P}f\left(\frac{P}{i_{FP}+P}\right)$, (A8)

and sea urchins ($U'$)

$U'=U+l_{U}^{ex}+l_{U}^{en}U+\kappa_{U}\theta_{U}(1-\lambda_{H}\theta_{H})\left(g_{M}M\mu_{M}+g_{T}T\mu_{T}+\zeta_{T}E\mu_{E}\right)-d_{U}U$. (A9)

The value of parameters defined in Table S1 are uniform across the four instantiations of the model for Bolinao. The value of exogenous recruitment for consumer groups differs between site (as detailed in Table S2), because these parameters were used to tune the model for each of the four instantiations. The range of exogenous recruitment for consumer groups in Bolinao were estimated from McManus et al. [4], and refined for each of the four sites through a process of model tuning during the simulation of historical reconstruction trajectories of the past 22 years (see Text S3). Larval supply is likely to vary between sites due to differences in hydrodynamic regimes present at different localities. Recruitment parameters are often used to tune models [5-7] because recruitment is inherently variable [8-11] and published estimates are uncertain and few.

**REFERENCES**

1. Fung T (2009) Local scale models of coral reef ecosystems for scenario testing and decision support. Phd thesis: University College London.

2. Fung T, Seymour RM, Johnson CR (2011) Alternative stable states and phase shifts in coral reefs under anthropogenic stress. Ecology 92: 967-982.

3. Melbourne-Thomas J, Johnson CR, Fung T, Seymour RM, Chérubin LM, et al. (2011) Regional-scale scenario modeling for coral reefs: a decision support tool to inform management of a complex system. Ecological Applications 21: 1380-1398.

4. McManus J, Nanola C, Reyes R, Kesner K (1992) Resource ecology of the Bolinao coral reef system. Manila, the Philippines: International Center for Living Aquatic Resources Management (ICLARM) Studies and Reviews 22.

5. Marzloff M, Shin Y, Tam J, Travers M, Bertrand A (2009) Trophic structure of the Peruvian marine ecosystem in 2000-2006: insights on the effects of management scenarios for the hake fishery using the IBM trophic model Osmose. Journal of Marine Systems 75: 290-304.

6. Melbourne-Thomas J, Johnson C, Fulton E (2011) Regional-scale scenario analysis for the Meso-American Reef system: Modelling coral reef futures under multiple stressors. Ecological Modelling 222: 1756-1770.

7. Melbourne-Thomas J, Johnson CR, Aliño PM, Geronimo RC, Villanoy CL, et al. (2011) A multi-scale biophysical model to inform regional management of coral reefs in the western Philippines and South China Sea. Environmental Modelling & Software 26: 66-82.

8. Richmond RH, Hunter CL (1990) Reproduction and recruitment of corals: comparisons among the Caribbean, the Tropical Pacific, and the Red Sea. Marine Ecology Progress Series 60: 185-203.

9. Sale PF, editor (1991) The Ecology of Fishes on Coral Reefs. London: Academic Press.

10. Doherty PJ (1991) Spatial and temporal patterns in recruitment. In: Sale PF, editor. The Ecology of Fishes on Coral Reefs. San Diego, California: Academic Press.

11. Valles H, Hunte W, Kramer DB (2009) Variable temporal relationships between environment and recruitment in coral reef fishes. Marine Ecology Progress Series 379: 225-240.
