# Supplementary material for: Modelling Coral Reef Futures to Inform Management: Can Reducing Local-Scale Stressors Conserve Reefs under Climate Change?
Source: PLoS One. 2013 Nov 18;8(11):e80137. doi: 10.1371/journal.pone.0080137 (PMC3832406; doi:10.1371/journal.pone.0080137)
Supplement: Text S2 — Additions to the model for Bolinao instantiation. (DOCX) [file pone.0080137.s011.docx]

**SUPPLEMENTARY MATERIAL**

**Text S2: Additions to the model for Bolinao instantiation**

**Scaling fish recruitment to the amount of coral cover:** In previous versions of the model [1], consumer groups interacted with benthic functional groups through grazing (on macroturf, EAC and macroalgae) only. Therefore, as the modeled reef becomes more degraded (i.e. coral is overgrown by macroturf and macroalgae), the biomass of sea urchins and herbivorous fish (and thus their predators, piscivores) increases in response to elevated algae. However, convincing evidence is emerging that the loss of coral cover on reefs may have a negative impact on all reef fish (including herbivores and piscivores) through the inhibition of recruitment [2-4]. Holbrook et al. [5] examined how recruitment changed over several levels of coral loss and found a positive asymptotic relationship between fish abundance and live coral cover, which was fitted to the Shepherd [6] function. Therefore, we added a term (denoted by$\delta$) to fish recruitment in the model to scale survival of recruits with coral cover (see Text S1). $\delta$ is equal to 0.25 when coral cover is < 5%, and 1 when coral cover (*C*) is > 60%. When 5 % ≤ *C* ≤ 60%, $\delta$ follows the Shepherd [6] function used by Holbrook et al. [5], *survival* = *aC*/[1 + (*a/b)C^d^*], where *C* is coral cover, and *a, b* and *d* are fitted parameters. We used *a* =101, *b* = 1.1 and *d* = 0.83.

**REFERENCES**

1. Fung T, Seymour RM, Johnson CR (2011) Alternative stable states and phase shifts in coral reefs under anthropogenic stress. Ecology 92: 967-982.

2. Wilson SK, Graham N, Pratchett M, Jones GB, Polunin N (2006) Multiple disturbances and the global degradation of coral reefs: are reef fishes at risk or resilient? Global Change Biology 12: 2220-2234.

3. Feary D, Almany G, McCormick MI, Jones GB (2007) Habitat choice, recruitment and the response of coral reef fishes to coral degradation. Oecologia 153: 727-737.

4. Pratchett M, Munday P, Wilson SK, Graham N, Cinner J, et al. (2008) Effects of climate-induced coral bleaching on coral-reef fishes-ecological and economic consequences. Oceanography and Marine Biology: An Annual Review 46: 251-296.

5. Holbrook SJ, Schmitt RJ, Brooks AJ (2008) Resistance and resilience of a coral reef fish community to changes in coral cover. Marine Ecology Progress Series 371: 263-271.

6. Shepherd J (1982) A versatile new stock-recruitment relationship for fisheries, and the construction of sustainable yield curves. ICES Journal of Marine Science 40: 67-75.
