# Supplementary material for: Modelling Coral Reef Futures to Inform Management: Can Reducing Local-Scale Stressors Conserve Reefs under Climate Change?
Source: PLoS One. 2013 Nov 18;8(11):e80137. doi: 10.1371/journal.pone.0080137 (PMC3832406; doi:10.1371/journal.pone.0080137)
Supplement: Text S3 — Model validation. (DOCX) [file pone.0080137.s012.docx]

**SUPPLEMENTARY MATERIAL**

**Text S3: Model validation**

To validate the model at each of the four sites we assessed whether the model could reproduce reef dynamics observed over the past 22 years, given a known series of stressors at these sites. We simulated historical trajectories of the last two decades and assessed whether model output corresponded with observations of reef state during that period. First, empirical values for state variables describing reef state at each site in 1987 were assembled (see Table S3 for summary of validation data and sources). Second, a historical timeline of stressors known to have affected the sites from 1987 – 2008 was constructed (Figure S1). Third, the model was run under a forcing schedule representing the historical timeline, and the average of 20 model runs was compared with empirical data of reef state (Figure S2, Figure S3).

Additional forcings (i.e. stressors) used in model validation

To undertake the model validation we added three additional stressors to the forcing scheme, namely typhoons, blast and poison fishing (Table S3), because these stressors were present during 1987 – 2008 in Bolinao.

Parameterization of the effect of blast and poison fishing on coral was derived from McManus et al. [1], who estimate that a rate of 10 blasts/hour (within an average listening radius of 2-3 km) corresponds to a 1.4% year^-1^ loss of existing cover of coral, and that poison fishing by forty people causes a 0.4% year^-1^ loss of existing coral. At a blasting rate of 10 blasts/hour, McManus et al. [2] report that blast fishing increased the total municipal fish yield at Bolinao, the Philippines, by 10 – 15%, and Calud et al. [3] estimate that at the same blasting rate, average municipal catch for the Lingayen Gulf (in which Bolinao is located) increased by 10%. Fish yields from poison fishing are considered relatively insignificant [2]. In the absence of any other data on the effects of blast and poison fishing, it is assumed that coral mortality and fishing yield decrease or increase linearly with rate of blasting and number of people practising poison fishing. Therefore, the blast fishing forcing implemented in the model acts to reduce coral cover by 0.14% year^-1^blast^-1^hour^-1^ and increase fishing pressure by 1% year^-1^blast^-1^hour^-1^. Similarly, the poison fishing forcing acts to reduce coral cover by 0.01% year^-1^ for each fisher using poison fishing.

Typhoons may damage coral reefs by reducing the cover of coral and macroalgae [4,5]. The magnitude of damage is highly variable depending on (a) properties of the typhoon (e.g. intensity, direction, size); (b) geography of the surrounding land and sea floor; (c) distance of the reef from the typhoon; and (d) the characteristics of the coral reef, e.g. coral species, size of colonies, macroalgae species [6-8]. Gardner et al. (2005) found that hurricanes reduce coral cover by ~17% on average in the Caribbean. Therefore, typhoons are modelled to reduce coral cover by 0.2 – 20%; a range is given to account for differences in species composition and local spatial variability in the susceptibility of sites to damage. While this is a fairly conservative range it is in keeping with observations that damage inflicted by typhoons on Bolinao reefs has been significantly less than the 60% loss of coral cover caused by the 1998 bleaching event (P.Alino pers comm., 2009). Furthermore, Bolinao’s reefs are characterised by structurally compact corals (e.g. massive *Porites* spp), which are more resistant to wave damage than branched forms of coral such as *Acropora* spp. The effect of typhoons on macroalgae is modelled as a 45 – 90% reduction in cover, in keeping with Mumby et al.’s [4] model of a Caribbean reef in which macroalgae cover is reduced by 90% under hurricanes. I extended this range to account for different species and hurricane intensities.

Historical timeline of stressors at Bolinao during 1987 – 2008

Modeled stressors (assumed to be uniform across all sites, in terms of their effect size and temporal occurrence) were bleaching, COTs outbreaks and destructive fishing. One major bleaching event has been recorded for Bolinao (in 1998), which we modeled as a 60% reduction in coral cover [9-11]. The only documented COTS outbreak occurred in 2006 and was modeled as reduction in coral cover by 9% (University of the Philippines, Marine Science Institute, unpublished data).

The rate of blast fishing in Bolinao was constant at 10 blasts/hour (within an average listening radius of 2-3 km) from 1987 till 1990, when there was a 90% reduction in blast fishing due to renewed enforcement of laws prohibiting destructive fishing [2,12]. Poison fishing, which was estimated to be undertaken by 40 fishers/year in Bolinao in the late 1980s [1,13], is assumed to have also decreased by 90% during 1990. Reports indicate continued decline of destructive fishing practises [14,15]; Spalding et al. [16] and Deocadez et al. [17] report there is currently little or no blast fishing in Bolinao. Therefore, blast fishing was modeled at a rate of 10 blasts/hour during 1987 – 1989, and 1 blast/hour during 1990 – 1992 (Figure S1). Similarly, poison fishing was modeled at a rate of 40 poison fishers/year during 1987 – 1989, and four poison fishers/year during 1990 – 1992.

Stressors in the timeline which vary between sites include fishing, typhoons, nutrification and sedimentation. Estimates of reef fish yield for Bolinao vary significantly. Campos et al. [18] and McManus et al. [2] both report a yield of 2.7 t km^-2^ year^-1^, while other studies report 5.56 t km^-2^ year^-1^ [19], 1.54 t km^-2^ year^-1^ [20] and 1.01 t km^-2^ year^-1^ [21]. Fish yield is reported to have stayed relatively constant since 1987 [2, P.Alino, pers comm., 2009]. Given the variability in estimates of fishing yield, we derived a range from the mid-range value for the model; 2.6 – 2.8 t km^-2^ year^-1^ and it is constant from 1987 – 2008 for all sites except Lucero. Lucero was made an MPA in 2004 and therefore at this site the fishing forcing acts in years 1987 – 2003 only. Fishing pressure acts on each fish functional group following Alino et al. [22], where 68.3% of the total catch on Bolinao’s reefs are herbivorous fish and 31.7% are piscivorous fish.

To determine which typhoons affected the Bolinao reef system during 1987 – 2008, the distance between Bolinao and the path of each typhoon was calculated using typhoon tracks data [23,24] and an algorithm to calculate distance between two geographic points [25]. Gardner et al. [26] defines the proximity at which typhoons can affect reefs (i.e. the zone of influence) as 65 km for category 1 and 2 typhoons, 90 km for category 3 typhoons and 130 km for category 4 and 5 typhoons. Bolinao was found to be within the zone of influence of four typhoons during 1987 – 2008 (Figure S1).

Quantitative data for water quality in Bolinao is scarce; there are only four published quantitative estimates of nutrient level at each site during 1987 – 2008 [27-30]. Thus, to estimate nutrient and sediment levels for the four sites during 1987 – 2008 it was necessary to make several assumptions and extrapolations. Using nutrient data from a water monitoring site in the Guiguiwanen Channel for 2002 – 2008 [31], we estimated the concentration of nitrogen and phosphate at Tomasa, Cangaluyan, Lucero and Malilnep by deriving a nutrient gradient for these sites from Villanueva et al. [28]. The estimated concentration of phosphate relative to the level at the Guiguiwanen Channel water monitoring site is 71% at Tomasa, 63% at Cangaluyan, 51% at Lucero and 38% at Malilnep. Similarly, the estimated concentration of ammonia relative to the level at the Guiguiwanen Channel water monitoring site is 52% at Tomasa, 45% at Cangaluyan, 38% at Lucero and 11% at Malilnep. Nutrification impacts are assumed at concentrations above a threshold of 0.5 μM DIN and 0.1 μM DIP. The derived estimates for all four sites are significantly greater than this threshold. Prior to 2002, for the period 1995 – 2002, concentrations of nitrogen and phosphate were estimated based on data from San Diego-McGlone et al. [32] by assuming that the percentage change in the concentration of these nutrients over this period was uniform across the Bolinao reef complex. Similarly, the temporal trend in the concentration of both nutrients during 1987 – 1995 was derived from David et al.’s [33] analysis of nutrient concentrations in sediment cores.

Only a single quantitative estimate of sedimentation rates for Bolinao during 1987 – 2008 was found in the literature; Wesseling et al. [34] reported sedimentation rates ranging from 10.5 – 30 mg cm^-2^day^-1^ with an average of 18.3 mg cm^-2^day^-1^. These rates are ‘high’ according to the criteria set by Rogers [35] and exceed the threshold (10  mg cm^-2^day^-1^) over which sedimentation is assumed in the model. In the absence of any other data we assumed that the stressors of sedimentation and nutrification occur together, which is often the case in coastal marine systems [36]. Therefore, in the 1987 – 2008 timeline, sedimentation occurs in the same years as nutrification.

Comparing simulated historical trajectories and known reef state

Model trajectories from 1987 – 2008 for each of the four sites showed reasonable correspondence with empirical observations of reef state at those sites (Figure S2, Figure S3). The model captured the steady decline in coral cover and increase in macroturf cover in Bolinao over the past 22 years. Loss of coral cover and subsequent increase in macroturf cover due to typhoons and bleaching events are evident in model trajectories, particularly in 1998 when Bolinao’s reefs were hit by a major bleaching event and Typhoon Babs (Figure S2). Coral cover did not recover following stressor events, suggesting the possibility that stressors such as nutrification and sedimentation, inhibited rehabilitation.

**REFERENCES**

1. McManus J, Reyes R, Nanola C (1997) Effects of some destructive fishing methods on coral cover and potential rates of recovery. Environmental Management 21: 69-78.

2. McManus J, Nanola C, Reyes R, Kesner K (1992) Resource ecology of the Bolinao coral reef system. Manila, the Philippines: International Center for Living Aquatic Resources Management (ICLARM) Studies and Reviews 22.

3. Calud A, Rodriguez J, Aruelo R, Aguilar G, Cinco E, et al. Preliminary results of a study of the municipal fisheries in Lingayen Gulf. In: Silvestre G, Miclat E, Chua T, editors; 1989; LaUnion. pp. 3-18.

4. Mumby PJ (2006) The impact of exploiting grazers (Scaridae) on the dynamics of Caribbean coral reefs. Ecological Applications 16: 747-769.

5. Cheal AJ, Coleman G, Delean S, Miller I, Osborne K, et al. (2002) Responses of coral and fish assemblages to a severe but short-lived tropical cyclone on the Great Barrier Reef, Australia. Coral Reefs 21: 131-142.

6. Puotinen M (2004) Tropical cyclone impacts on coral reef communities: modelling the disturbance regime in the Great Barrier Reef region, 1969-2003. Phd thesis. Townsville: James Cook University.

7. Wantiez L, Chateau O, Le Mouellic S (2006) Initial and mid-term impacts of cyclone Erica on coral reef fish communities and habitat in the South Lagoon Marine Park of New Caledonia. Journal of the Marine Biological Association of the United Kingdom 86: 1229-1236.

8. Treml E, Cogan M, Keevican M. Hurricane disturbance and coral reef development: a geographic information system (GIS) analysis of 501 years of hurricane data from the Lessar Antilles. ; 1997; Panama. pp. 541-546.

9. Cesar H, Pet-Soede L, Quibilan MCC, Alino P, Arceo H, et al. (2001) First evaluation of the impacts of the 1998 coral bleaching event to fisheries and tourism in the Philippines. In: Schuttenberg H, editor. Coral bleaching: Causes, consequences and responses (Selected papers presented at the 9th International Coral Reef Symposium, October 2000). Rhode Island, USA: Coastal Resources Center.

10. Pet-Soede L (2000) Effects of coral bleaching on the socio-economics of the fishery in Bolinao, Pangasinan, Philippines. MSc thesis. Manila: University of the Philippines.

11. Arceo H, Quibilan MCC, Alino P, Lim G, Licuanan WY (2001) Coral bleaching in Philippine reefs: coincident evidences with mesoscale thermal anomalies. Bulletin of Marine Science 69: 579-593.

12. McManus J, Nanola C, Reyes R (1991) Destructive coral reef fishing: seeking perspectives. Working Paper No 79 Fisheries Stock Assessment Title XII Collaborative Research Support Program. University of Rhode Island, Rhode Island.

13. Hingco T, Rivera R (1991) Aquarium fish industry in the Philippines: toward development or destruction? In: Chou L, Chua T, Khoo H, Wong P, editors. Towards an integrated management of tropical coastal resources ICLARM Conference Proceedings. Singapore. pp. 249-253.

14. Bryant D, Burke L, McManus J, Spalding MD (1998) Reefs at risk: a map-based indicator of threats to the worlds coral reefs. WRI/ICLARM/WCMC/UNEP. Washington, D.C.: World Resources Institute.

15. Licuanan WY, Gomez E (2000) Philippine coral reefs, reef fishes and associated fisheries: status and recommendations to improve their management. Manila: Global Coral Reef Monitoring Network (GCRMN) Report.

16. Spalding MD, Ravilious C, Green EP (2001) World Atlas of Coral Reefs. Berkeley: University of California Press.

17. Deocadez M, Alino P, Bautista A, Gaite P (2003) Lingayen Gulf, Northwestern Philippines. Manila: Coral Reef Information Network of the Philippines (Philreefs).

18. Campos W, Norte-Campos A, McManus J (1994) Yield estimates, catch, effort and fishery potential of the reef flat in Cape Bolinao, Philippines. Journal of Applied Ichthyology 10: 82-95.

19. Cruz-Trinidad A, Geronimo R, Alino P (2007) Understanding the coral reef ecology-economy linkage in Bolinao, Pangasinan. Manila: University of the Philippines.

20. SAGIP (2003) SAGIP Lingayen Gulf Project: Site profile of Bolinao. Marine Science Institute, University of the Philippines, Manila.

21. Estepa N, Salmo S, Alino P (2001) Fish landed catch assessment in Bolinao as baseline information for community-based coastal resource management. Manila: University of the Philippines Marine Science Institute.

22. Alino P, McManus L, McManus J, Nanola C, Fortes G, et al. (1993) Initial parameter estimations of a coral reef flat ecosystem in Bolinao, Pangasinan, northwestern Philippines. ICLRAM Conference Proceedings. 252-258

23. JTWC (2009) Joint Typhoon Warning Center Western North Pacific Best Track Data. <http://metocph.nmci.navy.mil/jtwc/best_tracks/wpindex.html>.

24. JMA (2009) Japan Meteorological Agency Regional Specialized Meterological Center Best Track Data. < http://www.jma.go.jp/jma/jma-eng/jma-center/rsmc-hp-pub-eg/besttrack.html>.

25. Vincenty T (1975) Direct and inverse solutions of geodesics on the ellipsoid with application of nested equations. Survey Review 23: 88-93.

26. Gardner TA, Cote IM, Gill JA, Grant A, Watkinson AR (2005) Hurricanes and Caribbean coral reefs: Impacts, recovery patterns, and role in long-term decline. Ecology 86: 174-184.

27. San Diego-McGlone M (2003) Water quality assessment of Lingayen Gulf. Philippine Scientist 40: 41-56.

28. Villanueva RD, Yap HT, Montano MNE (2006) Intensive fish farming in the Philippines is detrimental to the reef-building coral *Pocillopora damicornis*. Marine Ecology Progress Series 316: 165-174.

29. Padayao D, San Diego-McGlone M (2000) Nitrogen and phosphorus in coastal systems: focus on dissolved organic N and P. Science Diliman 12: 51-58.

30. Maaliw MA, Bermas N, Mercado R, Guarin F. Preliminary results of a water quality baseline study of Lingayen Gulf; 1989; Manila. International Center for Aquatic and Living Resources Management.

31. San Diego-McGlone M, Tentia M (2008) Monitoring of water quality parameters in Bolinao, Pangasinan. Manila: Marine Science Institute, University of the Philippines.

32. San Diego-McGlone M, Azanza R, Villanoy CL, Jacinto G (2008) Eutrophic waters, algal bloom and fish kill in fish farming areas in Bolinao, Pangasinan, Philippines. Marine Pollution Bulletin 57: 295-301.

33. David C, Maria Y, Siringan F, Reotita J, Zamora P, et al. (2008) Coastal pollution due to increasing nutrient flux in aquaculture sites. Environmental Geology 58: 447-454.

34. Wesseling E, Uychiaoco A, Alino P, Vermaat J (2001) Partial mortality in *Porites* corals: variation among Philippine reefs. International Review of Hydrobiology 86: 77-85.

35. Rogers CS (1990) Responses of coral reefs and reef organisms to sedimentation. Marine Ecology Progress Series 62: 185-202.

36. Dustan P (1998) Coral reefs: Harbingers of global change? In: Hatziolos M, Hooten AJ, Martin F, editors. Coral reefs: Challenges and opportunities for sustainable management. Washington, DC, USA: The World Bank. pp. 139-141.
